# Psychometric Properties and Factor Structure of the Patient Health Questionnaire-9 as a Screening Tool for Depression Among Ecuadorian College Students

**DOI:** 10.3389/fpsyg.2022.813894

**Published:** 2022-04-29

**Authors:** Víctor Manuel López-Guerra, Carla López-Núñez, Silvia L. Vaca-Gallegos, Pablo V. Torres-Carrión

**Affiliations:** ^1^Department of Psychology, Universidad Técnica Particular de Loja, Loja, Ecuador; ^2^Department of Personality, Assessment and Psychological Treatments, School of Psychology, University of Seville, Seville, Spain; ^3^Department of Computer Science, Universidad Técnica Particular de Loja, Loja, Ecuador

**Keywords:** psychometric properties, factor structure, Patient Health Questionnaire-9 (PHQ-9), depression, college students, Ecuador

## Abstract

**Background:**

The Patient Health Questionnaire-9 (PHQ-9) is the most well-known self-report measure to screen for depressive symptomatology, although discerning which is the factor structure that represents the best fit remains a challenge.

**Objective:**

The aim of this study was to analyze the psychometric properties and factorial structure of the Spanish version of the PHQ-9 in a large sample of Ecuadorian college students.

**Methods:**

A total of 5,394 students (*M* = 22.03 years; *SD* = 3.05; 54.8% female) from three Ecuadorian universities were surveyed using a computerized questionnaire within a 4-week assessment period. The PHQ-9 was tested for factorial structure, reliability, and correlations with other mental health-related measures.

**Results:**

The hierarchical model that included a general factor and three nested factors had a better fit, and such a hierarchical structure remained invariant across sex and age. Total omega value (ω) was 0.90 for the total scale and hierarchical omega values (ω*h*) were 0.68 for the general factor, as well as 0.34, 0.38, and 0.44 for the respective nested factors (somatic, cognitive/affective, concentration/motor). Moreover, PHQ-9 scores were significantly higher among women, although effect sizes of comparison of those sex differences were very small. Finally, the PHQ-9 scores correlated with multiple mental health indicators (psychological inflexibility, loneliness, stress, and satisfaction with life).

**Discussion:**

The Spanish version of the PHQ-9 showed good psychometric properties among Ecuadorian college students. The hierarchical structure showed the best fit for the data, including not only a dominant general factor, but also the somatic, cognitive/affective and concentration/motor subcomponents. In conclusion, the PHQ-9 tool seems to represent an easy-to-implement screening questionnaire to design preventive programs for those Ecuadorian college students at risk of developing depressive disorders.

## Introduction

Depression is a common psychological disorder affecting more than 280 million people worldwide, as well as a leading cause of disability and overall global burden of disease (World Health Organization, 2021). In particular, unipolar depression represents the fifth leading cause of illness and death in low- and middle-income countries (LMICs) (Lopez and Mathers, 2013; Schantz et al., 2017). Latin America is one of the regions with the highest rates of depression, including Ecuador (Pan American Health Organization, 2018), a country where previous research has already highlighted high rates of moderate and severe symptoms within its specific cultural context (Yusim et al., 2009; Kohn et al., 2018). Overall, this impairing mental health condition is the source of immense human suffering (Adewuya et al., 2006; World Health Organization, 2021), and persons with major depression have a greater chance of dying prematurely than the general population due to unattended physical health problems and suicide (World Health Organization, 2013, 2021). Therefore, treating depression through integrated mental health and social care services in community-based settings represents a key global target (World Health Organization, 2013).

Entering university represents a critical period for developing depression as moving from high school to college means a difficult and stressful life transition, as these “emerging adults” (Thomas et al., 2017) could face new challenges such as leaving their homes, living with other students, loneliness, as well as economic and social problems (Weitzman, 2004; Cleary et al., 2011). Therefore, psychological distress and maladaptive adjustment to college could lead to the emergence of several mental health problems, mainly anxiety and depression disorders (e.g., Eisenberg et al., 2007; Vanstone and Hicks, 2019). In particular, it seems that college students usually meet higher rates of depression in comparison to the general population (Ibrahim et al., 2013), leading to academic and social disadvantages (e.g., Harrington et al., 1993; Adewuya et al., 2006).

A recent systematic review and meta-analysis (Akhtar et al., 2020) highlighted the high prevalence of depression among college students in LMICs. In Ecuador, transition to university may be especially challenging due to some cultural factors such as moving from rural areas to cities for the first time, adaptating to new values and cultural practices (especially among Indigenous populations; Martín-Díaz, 2017) or poverty, among others. In this context, previous research has also shown that there is a high prevalence of depressive symptomatology among college students in Ecuador that predicts in turn other health problems such as substance use and abuse (Ruisoto et al., 2016; Torres et al., 2017).

Despite the fact that there are effective psychological and pharmacological treatments for moderate and severe depression (World Health Organization, 2021), mental health practitioners need validated instruments in all socio-cultural contexts to effectively detect such symptomatology across the population (including college students). Previous research has shown that 76–85% of people in LMICs receive no treatment for their disorder (Wang et al., 2007; Evans-Lacko et al., 2018; World Health Organization, 2021), and one of the major barriers to effective care include inaccurate assessment. In this line, World Health Organization (2021) states that people who are depressed are often not correctly diagnosed, and others who do not have such disorder are misdiagnosed and prescribed antidepressants. This concern is also common in the Ecuadorian cultural context, where there is a lack of validated tools to both measure mental health (in general) and depression (in particular) among adult populations.

Overall, screening and proper identification of depression are necessary to address this public health problem in Ecuador. As previous authors recommend (Schantz et al., 2017), resource-limited settings (as Ecuador) should rely on self-report screening tools that are easier to implement than other time intensive and costly diagnostic interviews. Among different validated self-report tools, the Patient Health Questionnaire-9 (PHQ-9) is one of the most widely used measures for the screening of depression worldwide, in both primary care settings and research studies (Mitchell et al., 2016; Lamela et al., 2020). The PHQ-9 represents an easy-to-implement 9-item depression screening tool (Kroenke et al., 2001) based on the DSM-IV (*Diagnostic and Statistical Manual of Mental Disorders IV-TR*; American Psychiatric Association, 2000) criteria for Major Depressive Disorder (MDD). This self-report questionnaire allows one to establish depressive disorder diagnoses and also grade the severity of depressive symptoms, from minimal to severe depression (Kroenke et al., 2001). Therefore, the PHQ-9 represents an efficient instrument that requires much less time of clinicians than the original PRIME-MD (Primary Care Evaluation of Mental Disorders) interviews (Spitzer et al., 1994, 1999).

The PHQ-9 not only has been used through clinical care settings and practice (e.g., Diez-Quevedo et al., 2001; Kroenke et al., 2001; Petersen et al., 2015; Beard et al., 2016; Guo et al., 2017; González-Blanch et al., 2018) but also among research studies with non-clinical populations (e.g., Familiar et al., 2015; Boothroyd et al., 2019; Patel et al., 2019; Villarreal-Zegarra et al., 2019; Arnold et al., 2020; Shin et al., 2020), including college student subsamples (e.g., Adewuya et al., 2006; Granillo, 2012; Cassiani-Miranda et al., 2017; Keum et al., 2018; Miranda and Scoppetta, 2018; Kim and Lee, 2019; Huarcaya-Victoria et al., 2020). The Spanish version of the PHQ-9 instrument has previously been validated across several contexts and countries, such as Chile (Baader et al., 2012; Borghero et al., 2018; Saldivia et al., 2019), Honduras (Wulsin et al., 2002), Mexico (Familiar et al., 2015), Puerto Rico (González-Rivera, 2019), Peru (Huarcaya-Victoria et al., 2020), or Spain (Diez-Quevedo et al., 2001; Pinto-Meza et al., 2005; González-Blanch et al., 2018). Moreover, some studies have found good reliability and validity of the PHQ-9 when applied to Spanish-speaking Latinos in the United States (e.g., Huang et al., 2006; Donlan and Lee, 2010; Merz et al., 2011; Granillo, 2012).

Despite the fact that the PHQ-9 represents a useful instrument for the screening of depressive symptomatology among both clinical and non-clinical samples, there is a lack of consensus in previous studies regarding its factor structure and measurement invariance (Doi et al., 2018). Firstly, preceding research has highlighted a unidimensional factor structure (e.g., Hansson et al., 2009; Patel et al., 2019; Villarreal-Zegarra et al., 2019), but other studies found a bifactorial structure (somatic and cognitive/affective factors) (e.g., Chilcot et al., 2013; Petersen et al., 2015; Beard et al., 2016; Guo et al., 2017; Arnold et al., 2020; Shin et al., 2020). This controversy is also present in studies carried out with college students, as some authors (e.g., Keum et al., 2018; Kim and Lee, 2019) found a clear unidimensional structure, whereas other researchers (Granillo, 2012; Cassiani-Miranda et al., 2017; Miranda and Scoppetta, 2018) described a two-dimensional factor structure. On the other hand, PHQ-9 measurement invariance is supported across different studies (Lamela et al., 2020), however, there are scarce studies regarding the sex measurement invariance of this scale, especially among college student subsamples (e.g., Keum et al., 2018). In this regard, it should be noted that Spanish versions of the PHQ-9 have also shown disparate data regarding the factor structure and measurement invariance across different Spanish cultural contexts. While a one-factor structure predominates in Argentina (Urtasun et al., 2019), Chile (Baader et al., 2012; Borghero et al., 2018; Saldivia et al., 2019), Honduras (Wulsin et al., 2002), Mexico (Familiar et al., 2015), Puerto Rico (González-Rivera, 2019), Spain (Diez-Quevedo et al., 2001; González-Blanch et al., 2018), and Latino communities in the United States (Huang et al., 2006; Donlan and Lee, 2010; Merz et al., 2011), other studies support a two-factor structure, as is the case in Colombia (Miranda and Scoppetta, 2018), Peru (Huarcaya-Victoria et al., 2020), and the United States (for Latin female college students; Granillo, 2012). Of these studies, only four assessed measurement invariance across the following variables: gender (González-Blanch et al., 2018; Miranda and Scoppetta, 2018); age, marital status, education level, and employment situation over time (González-Blanch et al., 2018); English and Spanish-speaking groups (Merz et al., 2011); and Latinas and non-Latina White female groups (Granillo, 2012). Due to this, it is necessary to increase awareness of the measurement invariance of the PHQ-9 across some key clinical and sociodemographic variables (such as sex or age).

Due to its clinical importance, the PHQ-9 could represent a useful screening tool to early detect those college students at risk of developing depressive disorders, as previous authors have already highlighted through non-clinical college populations (e.g., Adewuya et al., 2006; Granillo, 2012). Nonetheless, studies in Ecuador are scarce and no validated screening tools in the country allow researchers and clinicians to reliably measure depression among college students. Moreover, following international guidelines for adapting tests across different cultures (Hambleton and Zenisky, 2011; Muñiz et al., 2013), it is necessary to examine both the factor structure and sex measurement invariance of the PHQ-9 in the specific Ecuadorian cultural context. With the purpose of overcoming these gaps in the literature, the aim of this study was to evaluate the psychometric properties and factor structure of the PHQ-9 for the assessment of depressive symptomatology among a non-clinical subsample of Ecuadorian college students.

## Materials and Methods

### Participants and Procedure

College students from three universities in Ecuador were invited via email to participate in the study, and they then completed a computerized survey within the 4-week assessment period. The initial sample was made up of 5,996 students and 602 questionnaires were discarded due to being filled in randomly or being incomplete. The final sample included 5,394 participants who completed the entire questionnaire as well as reported a full academic year at any of the three institutions included (average response rate across universities was 47.80%, ranging from 39.10 to 56.10%). Mean age was 22.03 years (*SD* = 3.05; range from 17 to 58 years old), and 54.8% were women. Additionally, 50.9% of the participants included in the final sample came from public universities, 94.5% were single, and 78.9% were full-time students. Moreover, 90% of participants were *mestizo* (that is, mixed-raced), 5% were Indigenous, 1.7% were Afro-Ecuadorian, and 1.5% were White.

A descriptive cross-sectional study was conducted within the framework of a broader project of prediction of drug consumption among college students (CEPRA XII-2018-05) from three of the main universities in Ecuador: *Universidad Técnica Particular de Loja (UTPL)*, *Universidad Técnica del Norte (UTN)*, and *Universidad Politécnica Salesiana (UPS)*. The study was approved by the Ethics Committee for Research in Human Beings (*Comite de Ética de Investigación en Seres Humanos*, UTPL-CEISH, March 6, 2019) of the UTPL Ecuador (UTPL-DIS-2019-0088-O) and was conducted according to the principles expressed in the Declaration of Helsinki (World Medical Association, 2013). Digital informed consent was obtained from all participants, who received personalized feedback on the results of their assessment. Prior to the final application of the online survey, the linguistic and cultural adaptation of the original PHQ-9 instrument into Ecuadorian Spanish language was tested on 30 participants as a pilot trial to test for the clarity and comprehensiveness of the questionnaire (see Appendix A). Overall, translation and adaptation processes of the scale were developed following the International Test Commission Guidelines—Second Edition (Hambleton and Zenisky, 2011; Muñiz et al., 2013).

### Measures

Patient Health Questionnaire-9 (PHQ-9; Kroenke et al., 2001). This self-reported version of the PHQ is a 9-item depression module from the full PHQ (Spitzer et al., 1999) that assesses depressive symptomatology according to the DSM-IV criteria for Major Depressive Disorder (MDD). Participants had to fill out the questionnaire following the instruction of how they felt in the preceding 2 weeks with regards to different areas [anhedonia, sleep disturbance, fatigue, depressed mood, low self-esteem, concentration problems, psychomotor problems, appetite changes, and suicidal ideation; e.g., see Lamela et al. (2020)]. The PHQ-9 includes a 4-point scale (0 = “not at all”; 1 = “several days”; 2 = “more than half the days”; 3 = “nearly every day”) to measure severity of depression and its score ranges from 0 to 27. The global assessment of this scale represents the sum of the nine items’ scores and a higher score indicates a greater depression symptomatology. According to the authors of the original scale (Kroenke et al., 2001), scores can be used as a continuous assessment or as a diagnosis of *major depression* if 5 or more symptoms have been reported in the past 2 weeks at least “more than half the days,” and one of the symptoms is “anhedonia” or “depressed mood.” Besides, the authors recommend diagnosing *other depression* if 2–4 symptoms have occurred at least “more than half the days” in the past 2 weeks, and if “depressed mood” or “anhedonia” symptoms are selected. An example item is “Trouble falling or staying asleep, or sleeping too much.”

Acceptance and Commitment Questionnaire—II (AAQ-II; Bond et al., 2011). It is the most widely used general measure of psychological inflexibility and includes 7 items, to which participants must respond in a 7-point Likert-type scale, ranging from 1 = “never” to 7 = “always.” Scores of this scale range from 7 to 49, and higher scores indicate a tendency to act under the need to control or avoid aversive thoughts, memories, or feelings. The Spanish version of the AAQ-II (see Paladines-Costa et al., 2021) is characterized by a unifactorial structure that explains 66.87–70.03% of the total variance of the scale responses, a good internal consistency (α = 0.919 and ω = 0.928) and significant correlation with multiple health indicators (e.g., perceived stress). An example item would be “It seems like most people are handling their lives better than I am.” Medium to strong positive correlations were expected between the PHQ-9 and AAQ-II scores.

Loneliness Scale Revised- Short (UCLA-3; Hughes et al., 2004). This scale consists of a brief 3-item scale assessing the subjective feeling of loneliness, understood as the perception of less social support being available than desired. Scores range from 0 to 9, and participants should answer to a series of statements with 1 to 3 points (1 = “hardly ever,” 2 = “some of the time,” and 3 = “often”). Overall, higher scores indicate a greater feeling of loneliness or lack of social support. The internal consistency was good for both men (α = 0.75) and women (α = 0.84). The following item represents an example of this scale: “How often do you feel isolated from others?” Medium to strong positive correlations were expected between the PHQ-9 and UCLA-3 scores.

Perceived Stress Scale [PSS-10; Cohen et al., 1983; short version developed by Remor (2006)]. This 10-item scale evaluates the degree to which people perceive a lack of control in their daily lives. Scores range from 0 to 40 points, and participants should respond to a 5-point Likert-type scale, ranging from 0 (“never”) to 4 (“very often”). Higher scores indicate higher levels of psychological stress. This scale was both linguistic and culturally adapted in Ecuador by Ruisoto et al. (2020), finding good reliability (α = 0.85 and ω = 0.87), a bifactorial structure (which explains 56.99% of the total variance), as well as a satisfactory convergent validity with multiple health indicators (e.g., psychological inflexibility). An example item would be “In the last month, how often have you felt that you were unable to control important things in your life?” Medium to strong positive correlations were expected between the PHQ-9 and PSS-10.

Satisfaction with Life Scale [SWLS; Diener et al., 1985; Spanish adaptation conducted by Atienza et al. (2000)]. This 5-item instrument is aimed at assessing global cognitive judgments of one’s life satisfaction (not tapping related theoretical constructs such as positive affect or loneliness). The SWLS includes a 7-point Likert-type scale where answers range from 1 = “strongly disagree” to 7 = “strongly agree.” Overall ratings range from 5 to 35, and higher values indicate an increased satisfaction with one’s life. According to the authors of the original scale (Diener et al., 1985) some cut-offs could be followed as benchmarks: 5–9 = “extremely dissatisfied”; 10–14 = “dissatisfied”; 15–19 = “slightly dissatisfied”; 20 = “neutral”; 21–25 = “slightly satisfied”; 26–30 = “satisfied”; 31–35 = “extremely satisfied.” The psychometric properties of this unidimensional scale are adequate, being the internal consistency of the Spanish adaptation good (α = *0.84*). An example item is: “So far I have gotten the important things I want in life.” Medium to strong negative correlations were expected between the PHQ-9 and SWLS scores.

### Data Analyses

The statistical analyses were carried out using the IBM Statistical Package for the Social Sciences (SPSS) software (IBM Inc., Chicago, IL, United States; version 26.0), AMOS version 25.0 (IBM Inc., Armonk, NY, United States) and Bifactor Indices Calculator: A Microsoft Excel-Based Tool to Calculate Various Indices Relevant to Bifactor CFA Models (Dueber, 2017).

Firstly, Kolmogorov–Smirnov normality and Levene’s homoscedasticity tests were conducted to test basic assumptions. Based on the distribution of the scores, a multivariate analysis of variance (MANOVA) test was also developed, offering the advantage of reducing type I error by assessing the sex differences of all items in the same analysis. Effect sizes of principal comparisons of sex differences were calculated using eta squared (η^2^), taking into account values for small (0.01–0.05), medium (0.06–0.13) and large (0.14) effects (Cohen, 1988).

Secondly, the factorial structure was analyzed by performing a confirmatory factor analysis (CFA), using the maximum likelihood method. The estimators of the goodness-of-fit were the Bentler comparative fit index (CFI), the Tucker–Lewis index (TLI), the root mean square error of approximation (RMSEA), the standardized root mean squared residual (SRMR), the chi ratio squared (χ^2^) by degrees of freedom (CMIN/DF), the Akaike information criterion (AIC), and the Bayesian information criterion (BIC). Good fitness of the model was considered if CFI and TLI ≥ 0.95, RMSEA ≤ 0.06, SRMR ≤ 0.08, and CMIN/DF ≤ 3. AIC and BIC were used to evaluate alternative models with the smaller value in each case indicating the best fitting model.

**FIGURE 1:**
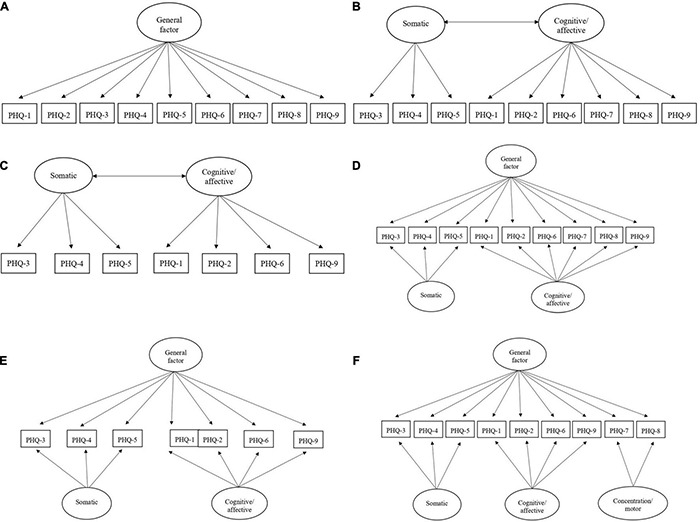
Tested models of the Ecuadorian version of PHQ-9. **(A)** *Model 1*. One-factor. **(B)** *Model 2*. Two-factors (somatic and cognitive/affective). **(C)** *Model 3*. Two-factors [somatic and cognitive/affective, but eliminating items 7 and 8; see Granillo (2012)]. **(D)** *Model 4*. Hierarchical structure that combines Models 1 and 2, including a general factor and two nested factors (somatic and cognitive/affective) (see Doi et al., 2018). **(E)** *Model 5*. Hierarchical structure that combines Models 1 and 3, eliminating items 7 and 8. **(F)** *Model 6*. Hierarchical structure that combines a general factor and three nested factors (somatic, cognitive/affective, and concentration/motor). PHQ-1, anhedonia; PHQ-2, depressed mood; PHQ-3, sleep difficulties; PHQ-4, fatigue; PHQ-5, appetite; PHQ-6, feeling of worthlessness; PHQ-7, concentration problems; PHQ-8, moving or speaking slowly; PHQ-9, suicidal ideation.

According to precedent literature using a CFA approach, we compared in our study the fit of six distinct and possible models of the Ecuadorian version of PHQ-9 (see Figure 1): *Model 1*, a unidimensional structure where each item is represented by a one-factor, therefore including the nine items assessed within a unique general factor (e.g., Baas et al., 2011; Yu et al., 2012; Bélanger et al., 2019); *Model 2*, a bidimensional model where the items are grouped into two-factors: somatic (includes three items, 3 = sleep difficulties, 4 = fatigue, 5 = appetite changes) and cognitive/affective (includes six items, 1 = anhedonia, 2 = depressed mood, 6 = feeling of worthlessness, 7 = concentration problems, 8 = moving or speaking slowly, 9 = suicidal ideation) (e.g., Patel et al., 2019; Lamela et al., 2020); *Model 3* also represents a two-factor model but eliminates items 7 and 8 (that is, the cognitive/affective dimension only includes the remaining four items) (see Granillo, 2012); *Model 4* represents a hierarchical structure that combines Models 1 and 2 as it includes two different nested factors (somatic and cognitive/affective) and a general factor that results from the sum of all item scores (see Doi et al., 2018); *Model 5* also represents a hierarchical structure, by combining Models 1 and 3, and removing items 7 and 8; and finally *Model 6*, a hierarchical structure that combines a general factor and three nested factors (somatic, cognitive/affective, and concentration/motor). It should be noted that loadings of the two-item factor concentration/motor have been constrained to be equal prior to standardization in order to achieve identifiability.

With the aim of assessing the hierarchical models, additional statistics such as ECV (Explained Common Variance), PUC (Percentage of Uncontaminated Correlations), replicability construct using H coefficient, omega coefficient (ω), and hierarchical omega coefficient (ω*h*) should be considered. Values greater than 0.70 for ECV and PUC support an essentially unidimensional structure of the PHQ-9 scale (Raykov and Hancock, 2005; Rodriguez et al., 2016). On the other hand, values included in the range 0.30–0.70 in ω*h*, H and ECV indexes reflect a substantive contribution of the factors or dimensions in explaining the construct variance; those values ≤ 0.30 indicate a poor contribution; and those values ≥ 0.70 indicate an excessive contribution (Moral et al., 2021).

Thirdly, we assessed the factorial invariance of the PHQ-9 across the total sample, taking into account the following models: configural invariance (*Model configural, MC*), which indicates a factorial structure without restrictions (baseline); metric invariance (*Model metric, MM*), where equivalence restrictions are established between factor loads; scalar invariance (*Model Scalar, SC*), that is, load and intercept equivalence restrictions; and strict invariance (*Model Strict, ST*), taking into account the equivalence restrictions of factor loads, intercepts, and residuals. Invariance tests for gender and age (<21 or >21 years old, that is, the median of the sample) were only planned for the best fitting model. We assessed the measurement invariance and its levels in accordance with the recommendations of Cheung and Rensvold (2002): ΔCFI ≤ 0.01 and ΔRMSEA ≤ 0.015.

Fourthly, internal consistency was analyzed based on omega coefficient (ω) and hierarchical omega coefficient (ω*h*), considering values ≥ 0.70 and 0.30 as satisfactory for ω and ω*h*, respectively. Finally, convergent and divergent validities were analyzed based on Pearson’s correlation (*r*) between the PHQ-9 scores and those scale’s ratings corresponding to different well-established psychological health indicators (psychological inflexibility, loneliness, perceived stress, and satisfaction with life). The size of Pearson’s correlation (*r*) values can range from 0.10 to 0.29 (*small*), 0.30 to 0.49 (*medium*), and 0.50 to 1.0 (*large*) (Cohen, 1988). In particular, *r* = 0.50–0.69 represents a *strong* value, *r* = 0.70–0.89 represents a *very strong* value, and ≥0.90 means that the relationship between the variables is *perfect* (Byrne, 2016).

## Results

### Total and Sex Differences in the Patient Health Questionnaire-9 in Ecuador

Firstly, we assessed whether the PHQ-9 scores differed by sex. Overall, female participants reported significantly higher scores for depression (*M* = 6.86; *SD* = 5.92) than male ones (*M* = 5.81; *SD* = 5.42) on the total score (*F* = 45.515; *p* < 0.001; η^2^ = 0.008). Such statistically significant differences were also found throughout most PHQ-9 items, except for items 8 (referring to “moving or speaking slowly”) and 9 (referring to “suicidal ideation”). Nonetheless, effect sizes of comparison of such sex differences were very small and insignificant (see Table 1).

**TABLE 1:** Sex differences in Patient Health Questionnaire-9 (PHQ-9) scores (item by item).

Items	Total sample (*N* = 5,394)	Female (*n* = 2,955)	Male (*n* = 2,439)	*F*	*p*	η^2^
				
	M (±SD)	M (±SD)	M (±SD)			
1. Little interest or pleasure in doing things	0.76 (±0.81)	0.80 (±0.821)	0.71 (±0.80)	15.886	<0.001	0.003
2. Feeling down, depressed, or hopeless	0.76 (±0.85)	0.85 (±0.86)	0.66 (±0.82)	68.595	<0.001	0.013
3. Trouble falling or staying asleep, or sleeping too much	0.83 (±0.92)	0.89 (±0.95)	0.76 (±0.87)	26.978	<0.001	0.005
4. Feeling tired or having little energy	1.04 (±0.87)	1.14 (±0.89)	0.92 (±0.84)	80.216	<0.001	0.015
5. Poor appetite or overeating	0.75 (±0.89)	0.84 (±0.93)	0.63 (±0.82)	75.578	<0.001	0.014
6. Feeling bad about yourself—or that you are a failure or have let yourself or your family down	0.73 (±0.90)	0.79 (±0.93)	0.66 (±0.85)	30.339	<0.001	0.006
7. Trouble concentrating on things, such as reading the newspaper or watching television	0.61 (±0.80)	0.65 (±0.83)	0.57 (±0.77)	13.924	<0.001	0.003
8. Moving or speaking so slowly that other people could have noticed? Or the opposite—being so fidgety or restless that you have been moving around a lot more than usual	0.52 (±0.75)	0.51 (±0.76)	0.53 (±0.73)	1.147	0.284	–
9. Thoughts that you would be better off dead or of hurting yourself in some way	0.39 (±0.74)	0.39 (±0.76)	0.37 (±0.73)	1.095	0.295	–
Total score	6.38 (±5.72)	6.86 (±5.92)	5.81 (±5.42)	45.515	<0.001	0.008

### Confirmatory Factorial Analysis

With the aim of determining the PHQ-9 factor structure, we compared the goodness-of-fit indexes of six distinct factor models of the Ecuadorian Spanish version of the scale (see Figure 1): one-factor structure (*Model 1*); bi-factor structure (somatic and cognitive/affective dimensions; *Model 2*); bi-factor structure (somatic and cognitive/affective dimensions) without items 7 and 8 (*Model 3*); hierarchical structure that combines Models 1 and 2 (*Model 4*); hierarchical structure that combines Models 1 and 3, and removing items 7 and 8 (*Model 5*); and hierarchical structure that combines a general factor and three nested factors (somatic, cognitive/affective, and concentration/motor) (*Model 6*).

The CFI, TIL, RMSEA, SRMR, and CMIN/DF goodness-of-fit indexes of *Models 1*–*5* were not as adequate as those presented by *Model 6* (CFI = 0.996; TLI = 0.947; RMSEA = 0.036; SRMR = 0.008 CMIN/DF = 8.185; AIC = 182.721; BIC = 380.562) (see Table 2), although that model showed higher values for both AIC and BIC indicators when compared to *Model 5* (AIC = 167.037; BIC = 167.102). Nevertheless, it is well-known that the AIC y BIC have explicit penalties for model complexity, and, in our analyses, Model 6 was definitely more complex that *Model 5.*

**TABLE 2:** Goodness-of-fit indexes for the confirmatory factor analysis (*n* = 5,394).

Models	CFI	TLI	RMSEA	SRMR	CMIN/DF	AIC	BIC
One-factor	0.960	0.947	0.086	0.023	36.892	1072.07	1150.75
Two-factor	0.977	0.968	0.063	0.016	22.616	626.02	751.29
Two-factor (without items 7 and 8)^b^	0.987	0.978	0.060	0.014	20.186	292.41	391.31
Hierarchical two nested factors and general factor^a^	0.988	0.975	0.055	0.013	17.552	369.93	547.94

Hierarchical two nested factors and general factor^a^	0.988	0.975	0.055	0.013	17.552	369.93	547.94
Hierarchical two nested factors and general factor (without items 7 and 8)	0.994	0.978	0.060	0.010	20.506	167.037	167.102
Hierarchical three nested factors and general factor	0.996	0.989	0.036	0.008	8.185	182.771	380.562

*^a^Model previously reported in Doi et al. (2018); Model previously reported in Granillo, 2012). CFI, Bentler comparative fit index; TLI, Tucker–Lewis index; RMSEA, root mean square error of approximation; SRMR, standardized root mean squared residual; CMIN/DF, ratio square (χ^2^) by degrees of freedom; AIC, Akaike information criterion; BIC, Bayesian information criterion.*

**FIGURE 2:**
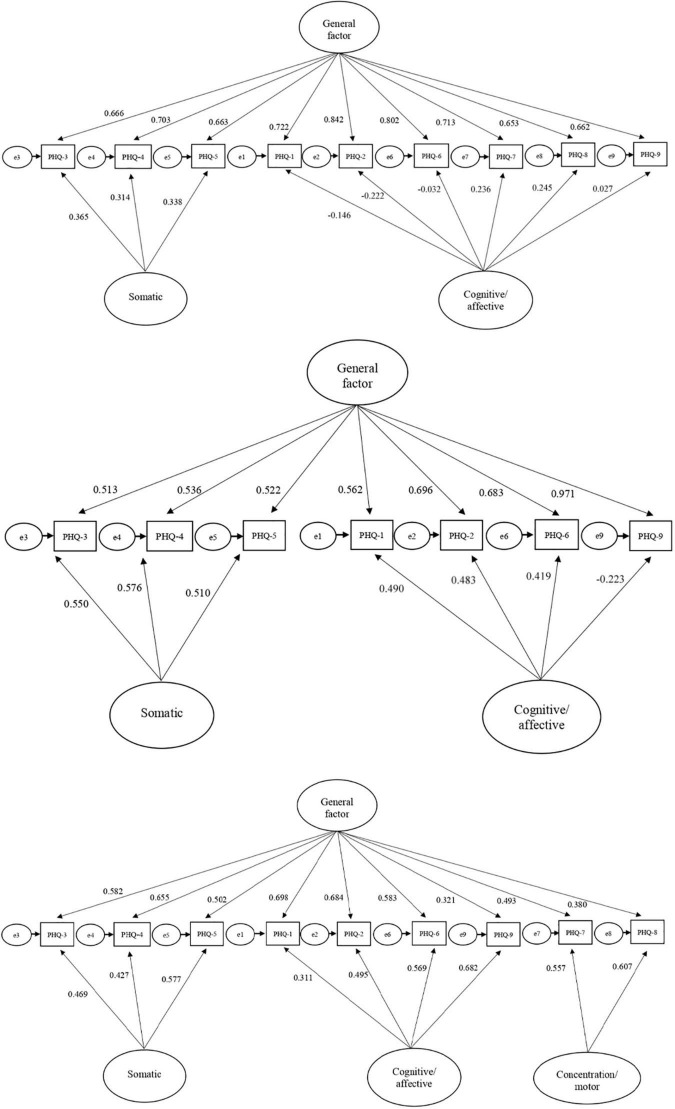
Patient Health Questionnaire-9 (PHQ-9) hierarchical models (*Model 4*, *Model 5*, and *Model 6*). *Model* 4. Hierarchical structure that combines Models 1 and 2, including two nested factors (somatic, cognitive/affective) and a general factor that results from the sum of all item scores (see Doi et al., 2018). *Model* 5. Hierarchical structure that combines Models 1 and 3, eliminating items 7 and 8. *Model* 6. Hierarchical structure that combines a general factor and three nested factors (somatic, cognitive/affective, and concentration/motor). PHQ-1, anhedonia; PHQ-2, depressed mood; PHQ-3, sleep difficulties; PHQ-4, fatigue; PHQ-5, appetite; PHQ-6, feeling of worthlessness; PHQ-7, concentration problems; PHQ-8, moving or speaking slowly; PHQ-9, suicidal ideation.

On the other hand, the additional assessment of the hierarchical models revealed that all factor loadings of *Model 6* were greater than 0.30 and positive, in contrast to *Models 4* and *5* (see Figure 2). The ECV values of the general and specific factors (see Table 3) suggested that *Model 4* is essentially unidimensional, progressively increasing such multidimensionality in hierarchical solutions 5 and 6. That is, the percentage of the variance explained by the nested factors increased and the one explained by the general factor decreased. Specifically, the general factor of *Model 6* explained the 52.8% of variance of depressive symptoms, and the three nested factors explained the 13.6, 21, and 12.6%, respectively. *Model 6* also showed satisfactory ω*h* and H indexes (ranging in the interval 0.30–0.70), suggesting a substantive contribution not only of the general factor but also of the nested factors in explaining the variance of depression. In addition, the increase in PUC (>0.70) in *Model 6* indicates that structural coefficients are relatively unbiased.

**TABLE 3:** Hierarchical model indexes (*n* = 5,394).

Models	Factor/nested factors	ECV	ω	Ω*h*	H	PUC
4. Hierarchical two nested factors and general factor	Somatic	0.067	0.802	0.161	0.282	0.500
	Cognitive/affective	0.036	0.883	0.001	0.165	
	General	0.896	0.917	0.894	0.913	
5. Hierarchical two nested factors and general factor (without items 7 and 8)	Somatic	0.192	0.798	0.413	0.559	0.571
	Cognitive/affective	0.151	0.901	0.125	0.470	
	General	0.657	0.910	0.759	0.952	
6. Hierarchical three nested factors and general factor	Somatic	0.136	0.807	0.337	0.501	0.722
	Cognitive/affective	0.210	0.872	0.379	0.640	
	Concentration/motor	0.126	0.694	0.444	0.508	
	General	0.528	0.900	0.684	0.822	

*ECV, Explained Common Variance; ω, omega coefficient; ωh, hierarchical omega coefficient; H, construct replicability index; PUC, Percentage of Uncontaminated Correlations.*

The adjustment indexes of the hierarchical *Model 6* (for both the total sample and separated by sex) are presented in Table 4, showing an adequate fit in each of them. On the one hand, the configural invariance (*MC*) presented good fit indicators (χ*^2^*(30) = 140.521; CFI = 0.995 and RMSEA = 0.026 [0.022–0.031]). Similarly, the metric invariance (*MM*) resulted on good fit indexes (χ*^2^*(44) = 182.527; CFI = 0.994; RMSEA = 0.024 [0.021–0.028]), being similar to the *MC* values since they showed minimal differences (ΔCFI = 0.001 and ΔRMSEA = −0.002). These results indicate that the factorial loads are invariant between the subsamples of men and women and, therefore, the covariances can be compared. On the other hand, the scalar invariance (SC) demonstrated indexes equal to the previous model (χ*^2^*(51) = 420.468; CFI = 0.993; RMSEA = 0.025 [0.022–0.028]) without minimal differences (ΔCFI = 0.001 and ΔRMSEA = 0.001), assuming the invariance between the different thresholds. Finally, the strict invariance (ST) reflects a good fit (χ*^2^*(60) = 262.937; CFI = 0.992; RMSEA = 0.025 [0.022–0.028]), showing no differences (ΔCFI = −0.001 and ΔRMSEA = 0) and verifying the invariance of the residuals. Overall, the combined results demonstrated the factorial invariance of the Ecuadorian version of the PHQ-9 across sex (both male and female college students) as well as across age (see Table 5).

**TABLE 4:** PQH-9 factorial invariance for the total sample and by sex.

Model	χ^2^	df	C-M	Δχ^2^	Δ*df*	CFI	ΔCFI	SRMR	RMSEA[CI 90%]	ΔRMSEA
Entire group	122.771	15	-	-	-	0.996	-	0.008	0.036 [0.031–0.043]	-
Men	54.729	15	-	-	-	0.996	-	0.008	0.033 [0.024–0.043]	-
Women	85.793	15	-	-	-	0.995	-	0.008	0.040 [0.032–0.048]	-
MC	140.521	30	-	-	-	0.995	-	0.008	0.026 [0.022–0.031]	-
MM	182.527	44	MM–MC	42.006	14	0.994	0.001	0.014	0.024 [0.021–0.028]	−0.002
SC	220.468	51	SC–MC	37.941	7	0.993	0.001	0.039	0.025 [0.022–0.028]	0.001
ST	262.937	60	ST–SC	42.469	9	0.992	−0.001	0.042	0.025 [0.022–0.028]	0

*χ^2^, chi-square analyses; df, degrees of freedom; C-M, comparison of factorial invariance models; CFI, Bentler comparative fit index; SRMR, standardized root mean squared residual; RMSEA, root mean square error of approximation, including its confidence intervals (CI = 90%); Δ, increase. Models: MC, Model Configural; MM, Model Metric; SC, Model Scalar; ST, Model Strict.*

**TABLE 5:** PQH-9 factorial invariance for the total sample and by age.

Model	χ^2^	df	C-M	Δχ^2^	Δ*df*	CFI	ΔCFI	SRMR	RMSEA [CI 90%]	ΔRMSEA
Entire group	122.771	15	-	-	-	0.996	-	0.008	0.036 [0.031–0.043]	-
≤21	79.255	15	-	-	-	0.995	-	0.009	0.038 [0.030–0.046]	-
≥22	59.981	15	-	-	-	0.996	-	0.007	0.036 [0.027–0.045]	-
MC	139.236	30	-	-	-	0.995	-	0.008	0.026 [0.022–0.030]	-
MM	158.259	44	MM–MC	19.023	14	0.995	0	0.010	0.022 [0.018–0.026]	−0.004
SC	194.561	51	SC–MC	36.302	7	0.994	−0.001	0.018	0.023 [0.019–0.026]	0.001
ST	366.790	60	ST–SC	172.29	9	0.987	−0.007	0.025	0.031 [0.028–0.034]	0.008

*χ^2^, chi-square analyses; df, degrees of freedom; C-M, comparison of factorial invariance models; CFI, Bentler comparative fit index; SRMR, standardized root mean squared residual; RMSEA, root mean square error of approximation, including its confidence intervals (CI = 90%); Δ, increase. Models: MC, Model Configural; MM, Model Metric; SC, Model Scalar; ST, Model Strict.*

### Internal Consistency and Convergent and Divergent Validities

The internal consistency of the hierarchical *Model 6* of the PHQ-9 scale showed satisfactory outcomes for both the total scale (ω = 0.90, ω*h* = 0.68) and its three nested factors: somatic (ω = 0.81, ω*h* = 0.34), cognitive/affective (ω = 0.87, ω*h* = 0.38) and concentration/motor (ω = 0.69, ω*h* = 0.44). Regarding convergent validity, we conducted correlation analyses between PHQ-9 scores and some of the most relevant mental health factors. The main results (see Table 6) showed positive and statistically significant relationships between depressive symptomatology scores and psychological inflexibility, loneliness, and perceived stress (*p* < 0.01). Convergent validity analyses also demonstrated negative and statistically significant associations between PHQ-9 scores and satisfaction with life (*p* < 0.01). Most of the effect sizes of PHQ-9 on the mental health indicators were moderate or large (*r* > 0.50).

**TABLE 6:** Correlation matrix between Patient Health Questionnaire-9 (PHQ-9) scores and other psychological health-related measures.

	Psychological inflexibility	Perceived stress	Loneliness	Satisfaction with life
PHQ-9 scores (depression)	0.690*	0.639*	0.581*	−0.479*

PHQ-9, Patient Health Questionnaire-9; *p < 0.01.

## Discussion

The main goal of the present study was to analyze the psychometric properties and factor structure of the PHQ-9 for the assessment of depression among a non-clinical subsample of Ecuadorian college students. To our knowledge, no research had previously evaluated both the psychometric properties and factor structure in the Ecuadorian culture. Overall, results showed the adequate psychometric properties of the Ecuadorian Spanish version of the scale and demonstrated that the bifactorial model had a better fit than the unidimensional did. Specifically, our findings not only support the presence of a dominant general factor but also suggest the existence of two distinct subcomponents (somatic and a cognitive/affective), remaining such a structure invariant across sex.

Firstly, results regarding depressive symptomatology across the sample showed low-to-middle scores, with females obtaining significantly higher scores compared to males. Previous studies have already highlighted that youth in LMICs are vulnerable to mental health issues (Pan American Health Organization, 2018; Akhtar et al., 2020) and Latin America is one of the regions with elevated rates of depressive symptomatology, including Ecuador among the top five countries that show higher rates of disability due to depression (Yusim et al., 2009; Pan American Health Organization, 2018). Moreover, our findings are in line with precedent research (e.g., Kuehner, 2017; World Health Organization, 2017; Tibubos et al., 2021) showing that depressive symptomatology is higher among females when compare to males, not only among the general population (e.g., Hyde et al., 2008; Parker and Brotchie, 2010; Salk et al., 2017) but also within the university context (Adewuya et al., 2006; Bitsika et al., 2010; Torres et al., 2017). In particular, depressive symptomatology in males could be related to violence, drug use and an increased risk of suffering somatic diseases, while women could express symptoms such as negative emotions, anhedonia or guilt, among others (Tibubos et al., 2021). In this vein, our results support precedent research since rates in females are significantly higher for most of the items of the PHQ-9 when compared to men (except for items 8 and 9). Nonetheless, it should be noted that effect sizes of such sex differences were very small in our study, although it is not strange considering that prevalence of depression not always differs between male and female students from LMICs (Akhtar et al., 2020). This result could be related to some barriers that female students are successfully overcoming, as their educational opportunities and rates of enrollment are gradually increasing (Akhtar et al., 2020). Beyond such small effect sizes, our results are important when taking into account that depression among college students is related to self-stigma (Waqas et al., 2014; Akhtar et al., 2020), poor academic performance (Adewuya et al., 2006; Akhtar et al., 2020), social disadvantages (Harrington et al., 1993), and financial difficulties (Andrews and Wilding, 2004), among others.

As previously reported in some studies (e.g., Huarcaya-Victoria et al., 2020), we did not find a good fit when considering a unique general factor (that is, a unidimensional structure) of the PHQ-9 scale. Due to this, other theoretical proposals (e.g., Granillo, 2012; Doi et al., 2018; Patel et al., 2019; Lamela et al., 2020) were empirically contrasted, showing that the hierarchical *Model 6* had the best fit to the data. In this sense, the Ecuadorian version of the PHQ-9 replicates previous results that supported a multidimensional structure of the scale, by including not only a general factor but also three nested factors (somatic, cognitive/affective, and concentration/motor) that allow researchers and clinicians to assess depressive symptomatology more accurately without disregarding a total score. In their recent systematic review of the PHQ-9 factor structure and measurement invariance, Lamela et al. (2020) stressed that there is no consensus on the factorial structure of this scale, ranging from one-factor to three-factor structures. In our study, the PHQ-9 scale clearly showed a higher hierarchical structure that contains three nested factors, as Marcos-Nájera et al. (2018) found among Spanish women in their first semester of pregnancy (cognitive-affective, somatic, and pregnancy-related), although it should be noted that not under a hierarchical model. In particular, the Ecuadorian version also showed a third nested factor by including items 7 and 8 as a separate entity (that is, *concentration problems* and *psychomotor disturbances*). We believe that such items are a separate dimension because they might have a different meaning for Ecuadorian college students. In this vein, Granillo (2012) excluded items 7 and 8 in her analyses and explained that some characteristics of college students (age, context) could lead to different interpretations of the activity levels and ability to focus on some tasks. This is not our case, as the best fit to the data occurred when both items were included and as separate entities. However, these results demonstrate that items 7 and 8 should be interpreted with caution when PHQ-9 is applied to college students, as the meaning of such items should be contextualized to their daily activities carried out within their role as students. For example, several studies have highlighted that, among college students, the decreased ability to concentrate symptom could be genuinely associated with higher levels of smartphone use, especially across campus spaces such as libraries, study rooms, etc. (Demirci et al., 2015; Wang et al., 2018).

Overall, while other studies offered no evidence for the necessity of using a multidimensional approach (e.g., Boothroyd et al., 2019), we believe that this multifactorial approach is relevant among Ecuadorian college students, as precedent studies have supported with both college students (Granillo, 2012; Cassiani-Miranda et al., 2017; Miranda and Scoppetta, 2018) and other subgroups of the population (Chilcot et al., 2013; Petersen et al., 2015; Beard et al., 2016; Guo et al., 2017; Doi et al., 2018; Arnold et al., 2020; Shin et al., 2020). This conclusion is important when taking into consideration culturally specific manifestations of depression and the social factors that influence them (Yusim et al., 2009). For example, somatic manifestations of depression seem important in previous research conducted in Latin American culture (Muñoz et al., 2005), and specifically in the Andean highlands of Ecuador (Yusim et al., 2009). These findings are similar to other studies where affective and somatic symptoms among Latino college students represent different subscales because the appearance of somatic symptomatology is considered the norm rather than the exception (e.g., Granillo, 2012).

Regarding measurement invariance, results of our study showed that the hierarchical structure remained invariant across men and women, supporting the idea that gender differences in the scores of the Ecuadorian version of the PHQ-9 are related to classical gender disparities in depression (Hyde et al., 2008; Parker and Brotchie, 2010; Salk et al., 2017) rather than the existence of bias in the measurement itself. Thus, we can conclude that the PHQ-9 scale can be equally administered across Ecuadorian female and male college students and, furthermore, it represents a sensitive assessment tool to existing gender differences in depressive symptomatology within an Ecuadorian context. Moreover, the symptoms assessed by the PHQ-9 scale are equivalent across different age groups. These findings are relevant as they allow us to compare male and female PHQ-9 scores as well as those results from groups of different ages, therefore replicating measurement invariance obtained in precedent research (e.g., Keum et al., 2018; Miranda and Scoppetta, 2018; Patel et al., 2019; Lamela et al., 2020; Tibubos et al., 2021). Overall, having a screening tool that is invariant across sex and different age groups will allow researchers and clinicians to develop both preventive and treatment programs of depression for both female and male college students in Ecuador.

On the other hand, our analyses revealed good internal consistency, not only for the total score but also for somatic, cognitive/affective, and concentration/motor scores. Such good reliability is similar to those results reported in precedent analyses of different PHQ-9 versions (e.g., Kroenke et al., 2001; Hansson et al., 2009; Doi et al., 2018; Huarcaya-Victoria et al., 2020; Lamela et al., 2020). Furthermore, the convergent and divergent validities of the Ecuadorian Spanish PHQ-9 showed that this depressive symptomatology correlated positively with other transdiagnostic health-related measures, such as psychological inflexibility (experiential avoidance or the tendency to suppress internal experiences; Hayes et al., 1996), loneliness (perceived social isolation; Cacioppo et al., 2006; Hawkley and Cacioppo, 2010), and perceived stress (perception of lack of control over the results of our behavior; Cohen et al., 1983); and negatively with satisfaction with life (global cognitive judgments of one’s life satisfaction; Diener et al., 1985). These findings were also consistent with previous studies, in which PHQ-9 scores were positively correlated with psychological inflexibility (Gilbert et al., 2019; Kato, 2020; McCracken et al., 2021; Paladines-Costa et al., 2021), psychological stress (Ruisoto et al., 2020), and perception of loneliness (Liu et al., 2020); and negatively correlated with satisfaction with life (Mei et al., 2021). The evidence of such associations is relevant as it supports the role of the PHQ-9 scale as a tool for the comprehensive assessment of mental health along with other key transdiagnostic variables.

Several limitations of this study merit mention. Firstly, our results should be considered with caution, since the sample only corresponds to college students. Precedent studies analyzing the factor structure of this scale did not have the limitation of only including college students and, in pursuit of overcoming this shortcoming, future research should further explore the psychometric properties and factor structure of the Ecuadorian Spanish version of PHQ-9 among other non-clinical and clinical populations as well as other age groups. Secondly, we only tested the measurement invariance among sex and different age subgroups, and it was not possible to assess other variables. For example, we were unable to evaluate measurement invariance across different ethnic groups, as most of the students were *mestizos* (that is, mixed-race). On the other hand, the range of ages has been limited, so future studies should analyze such measurement invariance across a wider age range as well as across other sociodemographic and clinical variables. Thirdly, this represents a cross-sectional study so measurement invariance, internal consistency as well as convergent and divergent validities should be replicated in future longitudinal designs. Finally, the use of a self-report tool (as the PHQ-9 scale) may be subject to some inaccuracies in data collection because of memory bias.

In spite of these shortcomings, our findings highlight the necessity of early identification of those Ecuadorian young people at risk of developing a serious psychological problem, as well as reveal that the PQH-9 could be a useful tool for the screening of depression among Ecuadorian college students. The analyses conducted clearly emphasize good psychometric properties of the Ecuadorian Spanish version of this scale and support a hierarchical model that includes not only a general factor but also three distinct nested factors (somatic, cognitive/affective, and concentration/motor), preserving measurement invariance across sex and age. Moreover, PHQ-9 scores correlate with other relevant transdiagnostic indicators of mental health, such as psychological inflexibility, loneliness, perceived stress, and satisfaction with life. These results are important as PHQ-9 represents an easy-to-implement screening questionnaire and a useful tool to develop preventive programs (Lamela et al., 2020), now including the Ecuadorian university context.

## Data Availability Statement

The raw data supporting the conclusions of this article will be made available by the authors, without undue reservation.

## Ethics Statement

The studies involving human participants were reviewed and approved by Ethics Committee for Research in Human Beings (Comité de Ética de Investigación en Seres Humanos, UTPL-CEISH, March 6, 2019) of the UTPL Ecuador (UTPL-DIS-2019-0088-O). Written informed consent from the participants’ legal guardian/next of kin was not required to participate in this study in accordance with the national legislation and the institutional requirements.

## Author Contributions

VL-G, CL-N, and SV-G: conceptualization, writing—original draft preparation, and writing—review and editing. VL-G: methodology, formal analysis, investigation, and data curation. VL-G and PT-C: supervision. PT-C and SV-G: project administration and funding acquisition. All authors have read and agreed to the published version of the manuscript.

## Conflict of Interest

The authors declare that the research was conducted in the absence of any commercial or financial relationships that could be construed as a potential conflict of interest.

## Publisher’s Note

All claims expressed in this article are solely those of the authors and do not necessarily represent those of their affiliated organizations, or those of the publisher, the editors and the reviewers. Any product that may be evaluated in this article, or claim that may be made by its manufacturer, is not guaranteed or endorsed by the publisher.

## APPENDIX A

**Table:**

Patient Health Questionnaire-9 (PHQ-9) adapted version to Ecuadorian Spanish from Kroenke et al. (2001)
1. Poco interés o placer en hacer cosas
2. Se ha sentido decaído(a), deprimido(a) o sin esperanzas
3. Ha tenido dificultad para quedarse o permanecer dormido(a), o ha dormido demasiado
4. Se ha sentido cansado(a) o con poca energía
5. Sin apetito o ha comido en exceso
6. Se ha sentido mal con usted mismo(a)—o que es un fracaso o que ha quedado mal con usted mismo(a) o con su familia
7. Ha tenido dificultad para concentrarse en ciertas actividades, tales como leer el periódico o ver la televisión
8. ¿Se ha movido o hablado tan lento que otras personas podrían haberlo notado? O lo contrario—muy inquieto(a) o agitado(a) o que ha estado moviéndose mucho más de lo normal
9. Pensamientos de que estaría mejor muerto(a) o de lastimarse de alguna manera
