# Supplementary material for: Psychometric Properties and Factor Structure of the Patient Health Questionnaire-9 as a Screening Tool for Depression Among Ecuadorian College Students
Source: Front Psychol. 2022 Apr 29;13:813894. doi: 10.3389/fpsyg.2022.813894 (PMC9105228; doi:10.3389/fpsyg.2022.813894)
Supplement: Supplementary file 1 [file Data_Sheet_1.PDF]

Supplementary material

MODEL 1: ONE-FACTOR

Standardized Regression Weights: (Total - Unconstrained)

			Estimate
ph9	<---	General	.649
ph8	<---	General	.635
ph7	<---	General	.696
ph6	<---	General	.802
ph2	<---	General	.822
ph1	<---	General	.721
ph5	<---	General	.700
ph4	<---	General	.737
ph3	<---	General	.704

CMIN

Model	NPAR	CMIN	DF	P	CMIN/DF
Default model	18	996.072	27	.000	36.892
Saturated model	45	.000	0		
Independence model	9	24262.637	36	.000	673.962

RMR, GFI

Model	RMR	GFI	AGFI	PGFI
Default model	.023	.957	.929	.574
Saturated model	.000	1.000		
Independence model	.334	.316	.145	.253

Baseline Comparisons

Model	NFI Delta1	RFI rho1	IFI Delta2	TLI rho2	CFI
Default model	.959	.945	.960	.947	.960
Saturated model	1.000		1.000		1.000
Independence model	.000	.000	.000	.000	.000

Parsimony-Adjusted Measures

Model	PRATIO	PNFI	PCFI
Default model	.750	.719	.720
Saturated model	.000	.000	.000
Independence model	1.000	.000	.000

NCP

Model	NCP	LO 90	HI 90
Default model	969.072	869.613	1075.928
Saturated model	.000	.000	.000
Independence model	24226.637	23717.551	24742.000

FMIN

Model	FMIN	F0	LO 90	HI 90
Default model	.185	.180	.161	.200
Saturated model	.000	.000	.000	.000
Independence model	4.499	4.492	4.398	4.588

RMSEA

Model	RMSEA	LO 90	HI 90	PCLOSE
Default model	.082	.077	.086	.000
Independence model	.353	.350	.357	.000

AIC

Model	AIC	BCC	BIC	CAIC
Default model	1032.072	1032.139	1150.747	1168.747
Saturated model	90.000	90.167	386.687	431.687
Independence model	24280.637	24280.670	24339.974	24348.974

ECVI

Model	ECVI	LO 90	HI 90	MECVI
Default model	.191	.173	.211	.191
Saturated model	.017	.017	.017	.017
Independence model	4.502	4.408	4.598	4.502

HOELTER

Model	HOELTER .05	HOELTER .01
Default model	218	255
Independence model	12	14

Minimization: .030

Miscellaneous: .828

Bootstrap: .000

Total: .858

Estimates (Group number 1 - Default model)

Scalar Estimates (Group number 1 - Default model)

Maximum Likelihood Estimates

Regression Weights: (Group number 1 - Default model)

			Estimate	S.E.	C.R.	P	Label
PHQ1	<---	F1	1.000				
PHQ2	<---	F1	1.194	.020	58.802	***	par_1
PHQ3	<---	F1	1.107	.022	49.642	***	par_2
PHQ4	<---	F1	1.100	.021	52.193	***	par_3
PHQ5	<---	F1	1.066	.022	49.059	***	par_4
PHQ6	<---	F1	1.232	.022	56.542	***	par_5
PHQ7	<---	F1	.957	.020	49.017	***	par_6
PHQ8	<---	F1	.814	.018	44.643	***	par_7
PHQ9	<---	F1	.827	.018	45.621	***	par_8

Standardized Regression Weights: (Group number 1 - Default model)

			Estimate
PHQ1	<---	F1	.721
PHQ2	<---	F1	.822
PHQ3	<---	F1	.704
PHQ4	<---	F1	.737
PHQ5	<---	F1	.700
PHQ6	<---	F1	.802
PHQ7	<---	F1	.696

	Estimate
PHQ8 <--- F1	.635
PHQ9 <--- F1	.649

Variances: (Group number 1 - Default model)

	Estimate	S.E.	C.R.	P	Label
F1	.344	.012	29.651	***	par_9
e1	.319	.007	46.413	***	par_10
e2	.236	.006	41.209	***	par_11
e3	.429	.009	46.836	***	par_12
e4	.351	.008	45.766	***	par_13
e5	.408	.009	46.928	***	par_14
e6	.291	.007	42.783	***	par_15
e7	.335	.007	47.132	***	par_16
e8	.338	.007	48.474	***	par_17
e9	.323	.007	48.169	***	par_18

Squared Multiple Correlations: (Group number 1 - Default model)

	Estimate
PHQ9	.421
PHQ8	.403
PHQ7	.484
PHQ6	.643
PHQ5	.490
PHQ4	.543
PHQ3	.496
PHQ2	.675
PHQ1	.519

Matrices (Group number 1 - Default model)

Implied (for all variables) Covariances (Group number 1 - Default model)

	F1	PHQ9	PHQ8	PHQ7	PHQ6	PHQ5	PHQ4	PHQ3	PHQ2	PHQ1
F1	.344									

	F1	PHQ9	PHQ8	PHQ7	PHQ6	PHQ5	PHQ4	PHQ3	PHQ2	PHQ1
PHQ9	.285	.559								
PHQ8	.280	.232	.566							
PHQ7	.329	.272	.268	.650						
PHQ6	.424	.351	.345	.406	.813					
PHQ5	.367	.304	.299	.351	.452	.799				
PHQ4	.378	.313	.308	.362	.466	.404	.767			
PHQ3	.381	.315	.310	.364	.469	.406	.419	.851		
PHQ2	.411	.340	.334	.393	.506	.438	.452	.455	.726	
PHQ1	.344	.285	.280	.329	.424	.367	.378	.381	.411	.663

Implied (for all variables) Correlations (Group number 1 - Default model)

	F1	PHQ9	PHQ8	PHQ7	PHQ6	PHQ5	PHQ4	PHQ3	PHQ2	PHQ1
F1	1.000									
PHQ9	.649	1.000								
PHQ8	.635	.412	1.000							
PHQ7	.696	.452	.442	1.000						
PHQ6	.802	.520	.509	.558	1.000					
PHQ5	.700	.454	.444	.487	.561	1.000				
PHQ4	.737	.478	.468	.513	.591	.515	1.000			

	F1	PHQ 9	PHQ 8	PHQ 7	PHQ 6	PHQ 5	PHQ 4	PHQ 3	PHQ 2	PHQ 1
PHQ 3	.704	.457	.447	.490	.564	.493	.519	1.000		
PHQ 2	.822	.534	.522	.572	.659	.575	.605	.578	1.000	
PHQ 1	.721	.468	.457	.501	.578	.504	.531	.507	.592	1.000

Implied Covariances (Group number 1 - Default model)

	PHQ 9	PHQ 8	PHQ 7	PHQ 6	PHQ 5	PHQ 4	PHQ 3	PHQ 2	PHQ 1
PHQ 9	.559								
PHQ 8	.232	.566							
PHQ 7	.272	.268	.650						
PHQ 6	.351	.345	.406	.813					
PHQ 5	.304	.299	.351	.452	.799				
PHQ 4	.313	.308	.362	.466	.404	.767			
PHQ 3	.315	.310	.364	.469	.406	.419	.851		
PHQ 2	.340	.334	.393	.506	.438	.452	.455	.726	
PHQ 1	.285	.280	.329	.424	.367	.378	.381	.411	.663

Implied Correlations (Group number 1 - Default model)

	PHQ 9	PHQ 8	PHQ 7	PHQ 6	PHQ 5	PHQ 4	PHQ 3	PHQ 2	PHQ 1
PHQ 9	1.000								
PHQ 8	.412	1.000							

	PHQ 9	PHQ 8	PHQ 7	PHQ 6	PHQ 5	PHQ 4	PHQ 3	PHQ 2	PHQ 1
PHQ 7	.452	.442	1.000						
PHQ 6	.520	.509	.558	1.000					
PHQ 5	.454	.444	.487	.561	1.000				
PHQ 4	.478	.468	.513	.591	.515	1.000			
PHQ 3	.457	.447	.490	.564	.493	.519	1.000		
PHQ 2	.534	.522	.572	.659	.575	.605	.578	1.000	
PHQ 1	.468	.457	.501	.578	.504	.531	.507	.592	1.000

Residual Covariances (Group number 1 - Default model)

	PHQ 9	PHQ 8	PHQ 7	PHQ 6	PHQ 5	PHQ 4	PHQ 3	PHQ 2	PHQ 1
PHQ 9	.000								
PHQ 8	.035	.000							
PHQ 7	.005	.051	.000						
PHQ 6	.033	-.008	.001	.000					
PHQ 5	-.024	.000	.008	-.002	.000				
PHQ 4	-.040	-.021	.000	-.021	.044	.000			
PHQ 3	-.039	-.009	-.014	-.015	.059	.051	.000		
PHQ 2	.022	-.014	-.018	.014	-.029	-.010	-.015	.000	
PHQ 1	-.019	-.011	-.010	-.009	-.031	.014	-.004	.037	.000

Standardized Residual Covariances (Group number 1 - Default model)

	PHQ9	PHQ8	PHQ7	PHQ6	PHQ5	PHQ4	PHQ3	PHQ2	PHQ1
PHQ9	.000								
PHQ8	4.178	.000							
PHQ7	.558	5.613	.000						
PHQ6	3.188	-.819	.069	.000					
PHQ5	-2.390	.005	.687	-.149	.000				
PHQ4	-4.060	-2.129	-.042	-1.686	3.675	.000			
PHQ3	-3.749	-.865	-1.245	-1.159	4.748	4.145	.000		
PHQ2	2.207	-1.463	-1.649	1.114	-2.412	-.810	-1.247	.000	
PHQ1	-2.065	-1.219	-.962	-.748	-2.807	1.305	-.384	3.366	.000

Factor Score Weights (Group number 1 - Default model)

	PHQ9	PHQ8	PHQ7	PHQ6	PHQ5	PHQ4	PHQ3	PHQ2	PHQ1
F1	.077	.073	.086	.128	.079	.094	.078	.153	.095

Total Effects (Group number 1 - Default model)

	F1
PHQ9	.827
PHQ8	.814
PHQ7	.957
PHQ6	1.232
PHQ5	1.066
PHQ4	1.100
PHQ3	1.107
PHQ2	1.194
PHQ1	1.000

Standardized Total Effects (Group number 1 - Default model)

	F1
PHQ9	.649
PHQ8	.635
PHQ7	.696
PHQ6	.802
PHQ5	.700
PHQ4	.737
PHQ3	.704
PHQ2	.822
PHQ1	.721

Direct Effects (Group number 1 - Default model)

	F1
PHQ9	.827
PHQ8	.814
PHQ7	.957
PHQ6	1.232
PHQ5	1.066
PHQ4	1.100
PHQ3	1.107
PHQ2	1.194
PHQ1	1.000

Standardized Direct Effects (Group number 1 - Default model)

	F1
PHQ9	.649
PHQ8	.635
PHQ7	.696
PHQ6	.802
PHQ5	.700
PHQ4	.737
PHQ3	.704
PHQ2	.822
PHQ1	.721

Indirect Effects (Group number 1 - Default model)

	F1
PHQ9	.000
PHQ8	.000
PHQ7	.000
PHQ6	.000
PHQ5	.000
PHQ4	.000
PHQ3	.000
PHQ2	.000
PHQ1	.000

Standardized Indirect Effects (Group number 1 - Default model)

	F1
PHQ9	.000
PHQ8	.000
PHQ7	.000
PHQ6	.000
PHQ5	.000
PHQ4	.000
PHQ3	.000
PHQ2	.000
PHQ1	.000

			M.I.	Par Change
e8	<-->	e9	66.766	.039
e7	<-->	e8	140.706	.058
e6	<-->	e9	78.161	.041
e6	<-->	e8	4.932	-.011
e5	<-->	e9	27.046	-.028
e4	<-->	e9	90.534	-.047
e4	<-->	e8	23.820	-.025
e4	<-->	e6	30.405	-.027
e4	<-->	e5	88.286	.053
e3	<-->	e9	67.585	-.045
e3	<-->	e7	8.726	-.017
e3	<-->	e6	12.526	-.019
e3	<-->	e5	128.813	.070
e3	<-->	e4	114.108	.062

	M.I.	Par Change
e2 <--> e9	42.856	.028
e2 <--> e8	17.981	-.018
e2 <--> e7	28.137	-.023
e2 <--> e6	21.370	.019
e2 <--> e5	61.090	-.038
e2 <--> e4	8.027	-.013
e2 <--> e3	16.593	-.020
e1 <--> e9	21.886	-.022
e1 <--> e8	7.300	-.013
e1 <--> e7	5.574	-.011
e1 <--> e6	5.585	-.011
e1 <--> e5	48.115	-.037
e1 <--> e4	12.094	.017
e1 <--> e2	129.412	.049

	M.I.	Par Change

	M.I.	Par Change
PHQ9 <--- PHQ8	37.685	.065
PHQ9 <--- PHQ6	23.845	.043
PHQ9 <--- PHQ5	12.725	-.032
PHQ9 <--- PHQ4	37.392	-.056
PHQ9 <--- PHQ3	31.365	-.049
PHQ9 <--- PHQ2	11.552	.032
PHQ9 <--- PHQ1	9.594	-.030
PHQ8 <--- PHQ9	36.321	.066
PHQ8 <--- PHQ7	66.978	.083
PHQ8 <--- PHQ4	9.832	-.029
PHQ8 <--- PHQ2	4.841	-.021
PHQ7 <--- PHQ8	79.526	.098
PHQ7 <--- PHQ3	4.058	-.018
PHQ7 <--- PHQ2	7.620	-.027
PHQ6 <--- PHQ9	42.877	.070
PHQ6 <--- PHQ4	12.729	-.033
PHQ6 <--- PHQ3	5.877	-.021
PHQ6 <--- PHQ2	5.910	.023
PHQ5 <--- PHQ9	14.742	-.047
PHQ5 <--- PHQ4	36.562	.063

	M.I.	Par Change
PHQ5 <--- PHQ3	59.911	.076
PHQ5 <--- PHQ2	16.552	-.043
PHQ5 <--- PHQ1	21.143	-.051
PHQ4 <--- PHQ9	49.429	-.080
PHQ4 <--- PHQ8	13.485	-.042
PHQ4 <--- PHQ6	9.357	-.029
PHQ4 <--- PHQ5	41.717	.062
PHQ4 <--- PHQ3	53.192	.067
PHQ4 <--- PHQ1	5.328	.024
PHQ3 <--- PHQ9	36.844	-.076
PHQ3 <--- PHQ7	4.165	-.024
PHQ3 <--- PHQ5	60.747	.081
PHQ3 <--- PHQ4	47.269	.073
PHQ3 <--- PHQ2	4.498	-.023
PHQ2 <--- PHQ9	23.566	.047
PHQ2 <--- PHQ8	10.247	-.031
PHQ2 <--- PHQ7	13.584	-.033
PHQ2 <--- PHQ6	6.705	.021
PHQ2 <--- PHQ5	29.146	-.044
PHQ2 <--- PHQ3	7.811	-.022
PHQ2 <--- PHQ1	57.636	.068
PHQ1 <--- PHQ9	11.940	-.037
PHQ1 <--- PHQ8	4.130	-.022
PHQ1 <--- PHQ5	22.712	-.043
PHQ1 <--- PHQ4	5.016	.021
PHQ1 <--- PHQ2	35.162	.056

Model	NPAR	CMIN	DF	P	CMIN/DF
Default model	18	996.072	27	.000	36.892
Saturated model	45	.000	0		
Independence model	9	24262.637	36	.000	673.962

Model	RMR	GFI	AGFI	PGFI
Default model	.023	.957	.929	.574
Saturated model	.000	1.000		
Independence model	.334	.316	.145	.253

Model	NFI Delta1	RFI rho1	IFI Delta2	TLI rho2	CFI
Default model	.959	.945	.960	.947	.960
Saturated model	1.000		1.000		1.000
Independence model	.000	.000	.000	.000	.000

Model	PRATIO	PNFI	PCFI
Default model	.750	.719	.720
Saturated model	.000	.000	.000
Independence model	1.000	.000	.000

Model	NCP	LO 90	HI 90
Default model	969.072	869.613	1075.928
Saturated model	.000	.000	.000
Independence model	24226.637	23717.551	24742.000

Model	FMIN	F0	LO 90	HI 90
Default model	.185	.180	.161	.200
Saturated model	.000	.000	.000	.000
Independence model	4.499	4.492	4.398	4.588

Model	RMSEA	LO 90	HI 90	PCLOSE
Default model	.082	.077	.086	.000
Independence model	.353	.350	.357	.000

Model	AIC	BCC	BIC	CAIC
Default model	1032.072	1032.139	1150.747	1168.747
Saturated model	90.000	90.167	386.687	431.687
Independence model	24280.637	24280.670	24339.974	24348.974

Model	ECVI	LO 90	HI 90	MECVI
Default model	.191	.173	.211	.191
Saturated model	.017	.017	.017	.017
Independence model	4.502	4.408	4.598	4.502

Model	HOELTER .05	HOELTER .01
Default model	218	255
Independence model	12	14

Minimization: .030

Miscellaneous: .828

Bootstrap: .000

Total: .858

MODEL 2: TWO-FACTORS

Model Fit Summary

CMIN

Model	NPAR	CMIN	DF	P	CMIN/DF
Default model	19	588.021	26	.000	22.616
Saturated model	45	.000	0		
Independence model	9	24262.637	36	.000	673.962

RMR, GFI

Model	RMR	GFI	AGFI	PGFI
Default model	.016	.975	.958	.564
Saturated model	.000	1.000		
Independence model	.334	.316	.145	.253

Baseline Comparisons

Model	NFI Delta1	RFI rho1	IFI Delta2	TLI rho2	CFI
Default model	.976	.966	.977	.968	.977
Saturated model	1.000		1.000		1.000
Independence model	.000	.000	.000	.000	.000

Parsimony-Adjusted Measures

Model	PRATIO	PNFI	PCFI
Default model	.722	.705	.705
Saturated model	.000	.000	.000
Independence model	1.000	.000	.000

NCP

Model	NCP	LO 90	HI 90
Default model	562.021	486.805	644.657
Saturated model	.000	.000	.000
Independence model	24226.637	23717.551	24742.000

FMIN

Model	FMIN	F0	LO 90	HI 90
Default model	.109	.104	.090	.120
Saturated model	.000	.000	.000	.000
Independence model	4.499	4.492	4.398	4.588

RMSEA

Model	RMSEA	LO 90	HI 90	PCLOSE
Default model	.063	.059	.068	.000
Independence model	.353	.350	.357	.000

AIC

Model	AIC	BCC	BIC	CAIC
Default model	626.021	626.091	751.288	770.288
Saturated model	90.000	90.167	386.687	431.687
Independence model	24280.637	24280.670	24339.974	24348.974

ECVI

Model	ECVI	LO 90	HI 90	MECVI
Default model	.116	.102	.131	.116
Saturated model	.017	.017	.017	.017
Independence model	4.502	4.408	4.598	4.502

HOELTER

Model	HOELTER .05	HOELTER .01
Default model	357	419
Independence model	12	14

Minimization:	.031
Miscellaneous:	.674
Bootstrap:	.000
Total:	.705

Estimates (Group number 1 - Default model)

Scalar Estimates (Group number 1 - Default model)

Maximum Likelihood Estimates

Regression Weights: (Group number 1 - Default model)

	Estimate	S.E.	C.R.	P	Label
PHQ3 <--- F1	1.000				
PHQ4 <--- F1	.989	.018	54.557	***	par_1
PHQ5 <--- F1	.960	.018	52.188	***	par_2
PHQ9 <--- F2	1.000				
PHQ8 <--- F2	.970	.023	42.103	***	par_3
PHQ7 <--- F2	1.136	.025	45.149	***	par_4
PHQ6 <--- F2	1.473	.029	51.480	***	par_5
PHQ2 <--- F2	1.432	.027	52.428	***	par_6
PHQ1 <--- F2	1.191	.026	46.349	***	par_7

Standardized Regression Weights: (Group number 1 - Default model)

	Estimate
PHQ3 <--- F1	.749
PHQ4 <--- F1	.780
PHQ5 <--- F1	.742
PHQ9 <--- F2	.662
PHQ8 <--- F2	.639
PHQ7 <--- F2	.697
PHQ6 <--- F2	.809
PHQ2 <--- F2	.832
PHQ1 <--- F2	.725

Covariances: (Group number 1 - Default model)

	Estimate	S.E.	C.R.	P	Label
F1 <--> F2	.308	.009	34.006	***	par_8

Correlations: (Group number 1 - Default model)

	Estimate
F1 <--> F2	.900

Variances: (Group number 1 - Default model)

	Estimate	S.E.	C.R.	P	Label
F1	.477	.016	30.343	***	par_9
F2	.245	.009	26.373	***	par_10
e1	.374	.009	41.240	***	par_11
e2	.301	.008	38.970	***	par_12
e3	.360	.009	41.738	***	par_13
e4	.314	.007	47.523	***	par_14
e5	.335	.007	48.017	***	par_15
e6	.334	.007	46.548	***	par_16
e7	.281	.007	41.336	***	par_17
e8	.223	.006	39.156	***	par_18
e9	.315	.007	45.724	***	par_19

Squared Multiple Correlations: (Group number 1 - Default model)

	Estimate
PHQ1	.525
PHQ2	.693
PHQ6	.655
PHQ7	.486
PHQ8	.408
PHQ9	.439
PHQ5	.550
PHQ4	.608
PHQ3	.560

Matrices (Group number 1 - Default model)

Implied (for all variables) Covariances (Group number 1 - Default model)

	F2	F1	PHQ1	PHQ2	PHQ6	PHQ7	PHQ8	PHQ9	PHQ5	PHQ4	PHQ3
F2	.245										
F1	.308	.477									
PHQ1	.292	.367	.663								
PHQ2	.351	.441	.418	.726							

	F2	F1	PHQ1	PHQ2	PHQ6	PHQ7	PHQ8	PHQ9	PHQ5	PHQ4	PHQ3
PHQ6	.361	.454	.430	.517	.813						
PHQ7	.278	.350	.332	.399	.410	.650					
PHQ8	.238	.299	.283	.341	.350	.270	.566				
PHQ9	.245	.308	.292	.351	.361	.278	.238	.559			
PHQ5	.296	.458	.352	.423	.435	.336	.287	.296	.799		
PHQ4	.305	.472	.363	.436	.449	.346	.295	.305	.453	.767	
PHQ3	.308	.477	.367	.441	.454	.350	.299	.308	.458	.472	.851

Implied (for all variables) Correlations (Group number 1 - Default model)

	F2	F1	PHQ1	PHQ2	PHQ6	PHQ7	PHQ8	PHQ9	PHQ5	PHQ4	PHQ3
F2	1.000										
F1	.900	1.000									
PHQ1	.725	.653	1.000								
PHQ2	.832	.750	.603	1.000							
PHQ6	.809	.729	.586	.674	1.000						
PHQ7	.697	.628	.505	.581	.564	1.000					
PHQ8	.639	.575	.463	.532	.517	.445	1.000				
PHQ9	.662	.596	.480	.551	.536	.462	.423	1.000			
PHQ5	.668	.742	.484	.556	.540	.466	.426	.442	1.000		

	F2	F1	PHQ1	PHQ2	PHQ6	PHQ7	PHQ8	PHQ9	PHQ5	PHQ4	PHQ3
PHQ4	.702	.780	.509	.584	.568	.490	.448	.465	.578	1.000	
PHQ3	.674	.749	.489	.561	.545	.470	.431	.446	.555	.584	1.000

Implied Covariances (Group number 1 - Default model)

	PHQ1	PHQ2	PHQ6	PHQ7	PHQ8	PHQ9	PHQ5	PHQ4	PHQ3
PHQ1	.663								
PHQ2	.418	.726							
PHQ6	.430	.517	.813						
PHQ7	.332	.399	.410	.650					
PHQ8	.283	.341	.350	.270	.566				
PHQ9	.292	.351	.361	.278	.238	.559			
PHQ5	.352	.423	.435	.336	.287	.296	.799		
PHQ4	.363	.436	.449	.346	.295	.305	.453	.767	
PHQ3	.367	.441	.454	.350	.299	.308	.458	.472	.851

Implied Correlations (Group number 1 - Default model)

	PHQ1	PHQ2	PHQ6	PHQ7	PHQ8	PHQ9	PHQ5	PHQ4	PHQ3
PHQ1	1.000								
PHQ2	.603	1.000							
PHQ6	.586	.674	1.000						

	PHQ 1	PHQ 2	PHQ 6	PHQ 7	PHQ 8	PHQ 9	PHQ 5	PHQ 4	PHQ 3
PHQ 7	.505	.581	.564	1.000					
PHQ 8	.463	.532	.517	.445	1.000				
PHQ 9	.480	.551	.536	.462	.423	1.000			
PHQ 5	.484	.556	.540	.466	.426	.442	1.000		
PHQ 4	.509	.584	.568	.490	.448	.465	.578	1.000	
PHQ 3	.489	.561	.545	.470	.431	.446	.555	.584	1.000

Residual Covariances (Group number 1 - Default model)

	PHQ 1	PHQ 2	PHQ 6	PHQ 7	PHQ 8	PHQ 9	PHQ 5	PHQ 4	PHQ 3
PHQ 1	.000								
PHQ 2	.029	.000							
PHQ 6	-.015	.003	.000						
PHQ 7	-.012	-.024	-.004	.000					
PHQ 8	-.015	-.021	-.014	.048	.000				
PHQ 9	-.026	.010	.023	-.001	.028	.000			
PHQ 5	-.016	-.014	.015	.023	.012	-.016	.000		
PHQ 4	.030	.006	-.003	.016	-.008	-.031	-.005	.000	
PHQ 3	.010	-.002	.001	.001	.002	-.031	.008	-.001	.000

Standardized Residual Covariances (Group number 1 - Default model)

	PHQ1	PHQ2	PHQ6	PHQ7	PHQ8	PHQ9	PHQ5	PHQ4	PHQ3
PHQ1	.000								
PHQ2	2.655	.000							
PHQ6	-1.294	.208	.000						
PHQ7	-1.214	-2.190	-.338	.000					
PHQ8	-1.582	-2.112	-1.338	5.355	.000				
PHQ9	-2.860	1.042	2.165	-.118	3.417	.000			
PHQ5	-1.489	-1.206	1.180	2.107	1.191	-1.600	.000		
PHQ4	2.762	.510	-.267	1.479	-.864	-3.201	-.411	.000	
PHQ3	.850	-.147	.048	.060	.223	-3.061	.605	-.098	.000

Factor Score Weights (Group number 1 - Default model)

	PHQ1	PHQ2	PHQ6	PHQ7	PHQ8	PHQ9	PHQ5	PHQ4	PHQ3
F2	.088	.150	.122	.079	.067	.074	.044	.054	.044
F1	.063	.106	.087	.056	.048	.053	.168	.207	.168

Total Effects (Group number 1 - Default model)

	F2	F1
PHQ1	1.191	.000
PHQ2	1.432	.000
PHQ6	1.473	.000
PHQ7	1.136	.000
PHQ8	.970	.000
PHQ9	1.000	.000
PHQ5	.000	.960
PHQ4	.000	.989
PHQ3	.000	1.000

Standardized Total Effects (Group number 1 - Default model)

	F2	F1
PHQ1	.725	.000
PHQ2	.832	.000
PHQ6	.809	.000
PHQ7	.697	.000
PHQ8	.639	.000
PHQ9	.662	.000
PHQ5	.000	.742
PHQ4	.000	.780
PHQ3	.000	.749

Direct Effects (Group number 1 - Default model)

	F2	F1
PHQ1	1.191	.000
PHQ2	1.432	.000
PHQ6	1.473	.000
PHQ7	1.136	.000
PHQ8	.970	.000
PHQ9	1.000	.000
PHQ5	.000	.960
PHQ4	.000	.989
PHQ3	.000	1.000

Standardized Direct Effects (Group number 1 - Default model)

	F2	F1
PHQ1	.725	.000
PHQ2	.832	.000
PHQ6	.809	.000
PHQ7	.697	.000
PHQ8	.639	.000
PHQ9	.662	.000
PHQ5	.000	.742
PHQ4	.000	.780
PHQ3	.000	.749

Indirect Effects (Group number 1 - Default model)

	F2	F1
PHQ1	.000	.000
PHQ2	.000	.000
PHQ6	.000	.000
PHQ7	.000	.000
PHQ8	.000	.000
PHQ9	.000	.000
PHQ5	.000	.000
PHQ4	.000	.000
PHQ3	.000	.000

Standardized Indirect Effects (Group number 1 - Default model)

	F2	F1
PHQ1	.000	.000
PHQ2	.000	.000
PHQ6	.000	.000
PHQ7	.000	.000
PHQ8	.000	.000
PHQ9	.000	.000
PHQ5	.000	.000
PHQ4	.000	.000
PHQ3	.000	.000

			M.I.	Par Change
e9	<-->	F2	7.174	-.007
e9	<-->	F1	9.938	.013
e8	<-->	e9	90.879	.040
e7	<-->	e9	18.214	-.020
e6	<-->	F2	13.416	-.010
e6	<-->	F1	18.533	.018
e6	<-->	e9	9.204	-.015
e6	<-->	e8	55.342	-.032
e5	<-->	e9	12.817	-.017
e5	<-->	e8	42.054	-.028
e5	<-->	e7	14.249	-.018
e5	<-->	e6	131.904	.057
e4	<-->	F2	56.873	.020
e4	<-->	F1	78.348	-.035

			M.I.	Par Change
e4	<-->	e9	45.146	-.031
e4	<-->	e8	11.048	.014
e4	<-->	e7	40.282	.029
e4	<-->	e5	47.582	.033
e3	<-->	e9	25.325	-.027
e3	<-->	e8	16.410	-.019
e3	<-->	e7	14.416	.020
e3	<-->	e6	17.985	.023
e3	<-->	e5	7.745	.015
e2	<-->	e9	47.961	.034
e2	<-->	e7	4.234	-.010
e2	<-->	e6	5.533	.012
e2	<-->	e5	7.412	-.014
e2	<-->	e4	34.285	-.028
e1	<-->	e4	20.413	-.024

	M.I.	Par Change

			M.I.	Par Change
PHQ1	<---	PHQ2	22.623	.045
PHQ1	<---	PHQ6	5.293	-.021
PHQ1	<---	PHQ7	4.353	-.021
PHQ1	<---	PHQ8	7.152	-.029
PHQ1	<---	PHQ9	23.682	-.053
PHQ1	<---	PHQ5	6.167	-.022
PHQ1	<---	PHQ4	20.479	.042
PHQ2	<---	PHQ1	39.859	.056
PHQ2	<---	PHQ7	26.572	-.046
PHQ2	<---	PHQ8	23.718	-.047
PHQ2	<---	PHQ9	5.867	.023
PHQ2	<---	PHQ5	7.426	-.022
PHQ6	<---	PHQ1	7.940	-.028
PHQ6	<---	PHQ8	8.007	-.030
PHQ6	<---	PHQ9	21.301	.049
PHQ6	<---	PHQ5	6.731	.023
PHQ7	<---	PHQ2	13.709	-.036
PHQ7	<---	PHQ8	73.505	.094
PHQ7	<---	PHQ5	11.894	.032

			M.I.	Par Change
PHQ7	<---	PHQ4	4.832	.021
PHQ8	<---	PHQ1	5.492	-.023
PHQ8	<---	PHQ2	10.338	-.031
PHQ8	<---	PHQ6	4.099	-.018
PHQ8	<---	PHQ7	62.099	.080
PHQ8	<---	PHQ9	24.866	.054
PHQ9	<---	F1	6.185	-.031
PHQ9	<---	PHQ1	19.367	-.043
PHQ9	<---	PHQ6	11.612	.030
PHQ9	<---	PHQ8	26.482	.054
PHQ9	<---	PHQ5	5.673	-.021
PHQ9	<---	PHQ4	25.860	-.046
PHQ9	<---	PHQ3	20.009	-.038
PHQ5	<---	PHQ1	11.193	-.037
PHQ5	<---	PHQ2	4.268	-.022
PHQ5	<---	PHQ7	8.118	.031
PHQ5	<---	PHQ8	4.088	.024
PHQ4	<---	PHQ1	22.980	.049
PHQ4	<---	PHQ9	15.886	-.045
PHQ3	<---	PHQ9	12.057	-.042

Iteration		Negative eigenvalues	Condition #	Smallest eigenvalue	Diameter	F	NTries	Ratio
0	e	4		-.746	9999.000	24192.048	0	9999.000
1	e	3		-.357	2.598	9093.345	20	.312
2	e	1		-.137	.399	5473.518	6	.953
3	e	1		-.003	.529	2412.793	5	.828
4	e	0	694.627		.474	1117.583	5	.798
5	e	0	199.523		.648	682.851	3	.000

Iteration		Negative eigenvalues	Condition #	Smallest eigenvalue	Diameter	F	NTries	Ratio
6	e	0	375.881		.199	591.307	1	1.086
7	e	0	432.887		.061	588.034	1	1.035
8	e	0	446.097		.004	588.021	1	1.004
9	e	0	446.453		.000	588.021	1	1.000

Model	NPAR	CMIN	DF	P	CMIN/DF
Default model	19	588.021	26	.000	22.616
Saturated model	45	.000	0		
Independence model	9	24262.637	36	.000	673.962

Model	RMR	GFI	AGFI	PGFI
Default model	.016	.975	.958	.564
Saturated model	.000	1.000		
Independence model	.334	.316	.145	.253

Model	NFI Delta1	RFI rho1	IFI Delta2	TLI rho2	CFI
Default model	.976	.966	.977	.968	.977
Saturated model	1.000		1.000		1.000
Independence model	.000	.000	.000	.000	.000

Model	PRATIO	PNFI	PCFI
Default model	.722	.705	.705
Saturated model	.000	.000	.000
Independence model	1.000	.000	.000

Model	NCP	LO 90	HI 90
Default model	562.021	486.805	644.657
Saturated model	.000	.000	

Model	NCP	LO 90	HI 90
			.000
Independence model	24226.637	23717.551	24742.000

Model	FMIN	F0	LO 90	HI 90
Default model	.109	.104	.090	.120
Saturated model	.000	.000	.000	.000
Independence model	4.499	4.492	4.398	4.588

Model	RMSEA	LO 90	HI 90	PCLOSE
Default model	.063	.059	.068	.000
Independence model	.353	.350	.357	.000

Model	AIC	BCC	BIC	CAIC
Default model	626.021	626.091	751.288	770.288
Saturated model	90.000	90.167	386.687	431.687
Independence model	24280.637	24280.670	24339.974	24348.974

Model	ECVI	LO 90	HI 90	MECVI
Default model	.116	.102	.131	.116
Saturated model	.017	.017	.017	.017
Independence model	4.502	4.408	4.598	4.502

Model	HOELTER .05	HOELTER .01
Default model	357	419
Independence model	12	14

Minimization:	.029
Miscellaneous:	.566
Bootstrap:	.000
Total:	.595

MODEL 3: TWO-FACTOR (WITHOUT ITEMS 7 AND 8)

Model Fit Summary

CMIN

Model	NPAR	CMIN	DF	P	CMIN/DF
Default model	15	262.413	13	.000	20.186
Saturated model	28	.000	0		
Independence model	7	18593.454	21	.000	885.403

RMR, GFI

Model	RMR	GFI	AGFI	PGFI
Default model	.014	.986	.969	.458
Saturated model	.000	1.000		
Independence model	.350	.362	.149	.271

Baseline Comparisons

Model	NFI Delta1	RFI rho1	IFI Delta2	TLI rho2	CFI
Default model	.986	.977	.987	.978	.987
Saturated model	1.000		1.000		1.000
Independence model	.000	.000	.000	.000	.000

Parsimony-Adjusted Measures

Model	PRATIO	PNFI	PCFI
Default model	.619	.610	.611
Saturated model	.000	.000	.000
Independence model	1.000	.000	.000

NCP

Model	NCP	LO 90	HI 90
Default model	249.413	200.450	305.809
Saturated model	.000	.000	.000
Independence model	18572.454	18127.148	19024.036

FMIN

Model	FMIN	F0	LO 90	HI 90
Default model	.049	.046	.037	.057
Saturated model	.000	.000	.000	.000
Independence model	3.448	3.444	3.361	3.528

RMSEA

Model	RMSEA	LO 90	HI 90	PCLOSE
Default model	.060	.053	.066	.005
Independence model	.405	.400	.410	.000

AIC

Model	AIC	BCC	BIC	CAIC
Default model	292.413	292.458	391.309	406.309
Saturated model	56.000	56.083	240.605	268.605
Independence model	18607.454	18607.475	18653.605	18660.605

ECVI

Model	ECVI	LO 90	HI 90	MECVI
Default model	.054	.045	.065	.054
Saturated model	.010	.010	.010	.010
Independence model	3.450	3.368	3.534	3.450

HOELTER

Model	HOELTER .05	HOELTER .01
Default model	460	570
Independence model	10	12

Minimization: .031
Miscellaneous: .606
Bootstrap: .000
Total: .637

Estimates (Group number 1 - Default model)

Scalar Estimates (Group number 1 - Default model)

Maximum Likelihood Estimates

Regression Weights: (Group number 1 - Default model)

			Estimate	S.E.	C.R.	P	Label
PHQ3	<---	F1	1.000				
PHQ4	<---	F1	.989	.018	54.052	***	par_1
PHQ5	<---	F1	.952	.018	51.534	***	par_2
PHQ9	<---	F2	1.000				
PHQ6	<---	F2	1.495	.030	49.998	***	par_3
PHQ2	<---	F2	1.487	.029	51.398	***	par_4
PHQ1	<---	F2	1.221	.027	45.384	***	par_5

Standardized Regression Weights: (Group number 1 - Default model)

	Estimate
PHQ3 <--- F1	.751
PHQ4 <--- F1	.782
PHQ5 <--- F1	.737
PHQ9 <--- F2	.653
PHQ6 <--- F2	.809
PHQ2 <--- F2	.852
PHQ1 <--- F2	.732

Covariances: (Group number 1 - Default model)

	Estimate	S.E.	C.R.	P	Label
F1 <--> F2	.300	.009	33.338	***	par_6

Correlations: (Group number 1 - Default model)

	Estimate
F1 <--> F2	.887

Variances: (Group number 1 - Default model)

	Estimate	S.E.	C.R.	P	Label
F1	.480	.016	30.305	***	par_7
F2	.238	.009	25.673	***	par_8
e1	.371	.009	40.487	***	par_9
e2	.298	.008	38.020	***	par_10
e3	.365	.009	41.401	***	par_11
e4	.321	.007	47.032	***	par_12
e7	.281	.007	38.877	***	par_13
e8	.199	.006	33.966	***	par_14
e9	.308	.007	44.291	***	par_15

Squared Multiple Correlations: (Group number 1 - Default model)

	Estimate
PHQ1	.536
PHQ2	.725
PHQ6	.654
PHQ9	.426

	Estimate
PHQ5	.543
PHQ4	.611
PHQ3	.564

Matrices (Group number 1 - Default model)

Implied (for all variables) Covariances (Group number 1 - Default model)

	F2	F1	PHQ1	PHQ2	PHQ6	PHQ9	PHQ5	PHQ4	PHQ3
F2	.238								
F1	.300	.480							
PHQ1	.291	.366	.663						
PHQ2	.354	.446	.432	.726					
PHQ6	.356	.448	.435	.529	.813				
PHQ9	.238	.300	.291	.354	.356	.559			
PHQ5	.285	.456	.348	.424	.426	.285	.799		
PHQ4	.296	.474	.362	.441	.443	.296	.451	.767	
PHQ3	.300	.480	.366	.446	.448	.300	.456	.474	.851

Implied (for all variables) Correlations (Group number 1 - Default model)

	F2	F1	PHQ 1	PHQ 2	PHQ 6	PHQ 9	PHQ 5	PHQ 4	PHQ 3
F2	1.00 0								
F1	.887	1.00 0							
PHQ 1	.732	.649	1.000						
PHQ 2	.852	.755	.623	1.000					
PHQ 6	.809	.717	.592	.689	1.000				
PHQ 9	.653	.579	.478	.556	.528	1.000			
PHQ 5	.654	.737	.478	.557	.529	.427	1.000		
PHQ 4	.693	.782	.508	.590	.561	.453	.576	1.000	

	F2	F1	PHQ 1	PHQ 2	PHQ 6	PHQ 9	PHQ 5	PHQ 4	PHQ 3
PHQ 3	.666	.751	.487	.567	.538	.435	.553	.587	1.000

Implied Covariances (Group number 1 - Default model)

	PHQ1	PHQ2	PHQ6	PHQ9	PHQ5	PHQ4	PHQ3
PHQ1	.663						
PHQ2	.432	.726					
PHQ6	.435	.529	.813				
PHQ9	.291	.354	.356	.559			
PHQ5	.348	.424	.426	.285	.799		
PHQ4	.362	.441	.443	.296	.451	.767	
PHQ3	.366	.446	.448	.300	.456	.474	.851

Implied Correlations (Group number 1 - Default model)

	PHQ1	PHQ2	PHQ6	PHQ9	PHQ5	PHQ4	PHQ3
PHQ1	1.000						
PHQ2	.623	1.000					
PHQ6	.592	.689	1.000				
PHQ9	.478	.556	.528	1.000			
PHQ5	.478	.557	.529	.427	1.000		
PHQ4	.508	.590	.561	.453	.576	1.000	
PHQ3	.487	.567	.538	.435	.553	.587	1.000

Residual Covariances (Group number 1 - Default model)

	PHQ1	PHQ2	PHQ6	PHQ9	PHQ5	PHQ4	PHQ3
PHQ1	.000						
PHQ2	.015	.000					
PHQ6	-.019	-.009	.000				
PHQ9	-.025	.007	.028	.000			
PHQ5	-.012	-.015	.024	-.005	.000		
PHQ4	.031	.002	.002	-.023	-.004	.000	
PHQ3	.011	-.006	.006	-.023	.009	-.004	.000

Standardized Residual Covariances (Group number 1 - Default model)

	PHQ1	PHQ2	PHQ6	PHQ9	PHQ5	PHQ4	PHQ3
PHQ1	.000						
PHQ2	1.371	.000					
PHQ6	-1.662	-.730	.000				
PHQ9	-2.721	.745	2.680	.000			
PHQ5	-1.131	-1.256	1.934	-.553	.000		
PHQ4	2.849	.130	.194	-2.381	-.290	.000	
PHQ3	.933	-.515	.496	-2.267	.723	-.301	.000

Factor Score Weights (Group number 1 - Default model)

	PHQ1	PHQ2	PHQ6	PHQ9	PHQ5	PHQ4	PHQ3
F2	.103	.194	.139	.081	.046	.058	.047
F1	.070	.131	.093	.055	.174	.221	.180

Total Effects (Group number 1 - Default model)

	F2	F1
PHQ1	1.221	.000
PHQ2	1.487	.000
PHQ6	1.495	.000
PHQ9	1.000	.000
PHQ5	.000	.952
PHQ4	.000	.989
PHQ3	.000	1.000

Standardized Total Effects (Group number 1 - Default model)

	F2	F1
PHQ1	.732	.000
PHQ2	.852	.000
PHQ6	.809	.000
PHQ9	.653	.000
PHQ5	.000	.737
PHQ4	.000	.782
PHQ3	.000	.751

Direct Effects (Group number 1 - Default model)

	F2	F1
PHQ1	1.221	.000
PHQ2	1.487	.000
PHQ6	1.495	.000
PHQ9	1.000	.000
PHQ5	.000	.952
PHQ4	.000	.989
PHQ3	.000	1.000

Standardized Direct Effects (Group number 1 - Default model)

	F2	F1
PHQ1	.732	.000
PHQ2	.852	.000
PHQ6	.809	.000
PHQ9	.653	.000
PHQ5	.000	.737
PHQ4	.000	.782
PHQ3	.000	.751

Indirect Effects (Group number 1 - Default model)

	F2	F1
PHQ1	.000	.000
PHQ2	.000	.000
PHQ6	.000	.000
PHQ9	.000	.000
PHQ5	.000	.000
PHQ4	.000	.000
PHQ3	.000	.000

Standardized Indirect Effects (Group number 1 - Default model)

	F2	F1
PHQ1	.000	.000
PHQ2	.000	.000
PHQ6	.000	.000
PHQ9	.000	.000
PHQ5	.000	.000
PHQ4	.000	.000

	F2	F1
PHQ3	.000	.000

	M.I.	Par Change
e9 <--> F2	10.532	-.009
e9 <--> F1	14.839	.016
e8 <--> F2	4.774	.005
e8 <--> F1	7.016	-.010
e8 <--> e9	31.159	.023
e7 <--> F2	9.531	-.008
e7 <--> F1	13.695	.015
e7 <--> e9	32.350	-.027
e7 <--> e8	13.289	-.015
e4 <--> F2	23.781	.014
e4 <--> F1	33.172	-.024
e4 <--> e9	42.002	-.031
e4 <--> e8	6.806	.011
e4 <--> e7	62.115	.037
e3 <--> e9	17.415	-.022
e3 <--> e8	14.014	-.018
e3 <--> e7	34.831	.031
e2 <--> e9	47.988	.034
e2 <--> e4	21.387	-.023
e1 <--> e4	11.433	-.018
e1 <--> e3	4.800	.013

	M.I.	Par Change

	M.I.	Par Change
PHQ1 <--- PHQ2	6.440	.024
PHQ1 <--- PHQ6	9.199	-.027
PHQ1 <--- PHQ9	22.430	-.051
PHQ1 <--- PHQ4	22.093	.043
PHQ2 <--- PHQ1	13.377	.032
PHQ2 <--- PHQ6	4.028	-.016
PHQ2 <--- PHQ5	8.450	-.023
PHQ6 <--- PHQ1	13.614	-.037
PHQ6 <--- PHQ9	33.509	.063
PHQ6 <--- PHQ5	19.866	.040
PHQ9 <--- PHQ1	17.250	-.041

			M.I.	Par Change
PHQ9	<---	PHQ6	17.413	.037
PHQ9	<---	PHQ4	14.789	-.035
PHQ9	<---	PHQ3	10.674	-.028
PHQ5	<---	PHQ1	8.283	-.032
PHQ5	<---	PHQ6	8.221	.029
PHQ4	<---	PHQ1	22.967	.050
PHQ4	<---	PHQ9	9.240	-.034
PHQ3	<---	PHQ9	6.895	-.032

Iteration		Negative eigenvalues	Condition #	Smallest eigenvalue	Diameter	F	NTries	Ratio
0	e	4		-.509	9999.000	19164.828	0	9999.000
1	e	2		-.253	2.078	6104.119	20	.509
2	e	0	6473.970		.378	3509.339	6	.782
3	e	0	219.036		.869	2283.100	6	.000
4	e	0	216.268		.744	970.880	2	.000
5	e	0	288.166		.154	340.338	1	1.181
6	e	0	314.208		.071	264.523	1	1.098
7	e	0	314.621		.014	262.416	1	1.024
8	e	0	315.311		.001	262.413	1	1.001

	par_1	par_2	par_3	par_4	par_5	par_6	par_7	par_8	par_9	par_10	par_11	par_12	par_13	par_14	par_15
par_1	.000														

	par_1	par_2	par_3	par_4	par_5	par_6	par_7	par_8	par_9	par_10	par_11	par_12	par_13	par_14	par_15
par_2	.000	.000													
par_3	.000	.000	.001												
par_4	.000	.000	.001	.001											
par_5	.000	.000	.001	.001	.001										
par_6	.000	.000	.000	.000	.000	.000									
par_7	.000	.000	.000	.000	.000	.000	.000								
par_8	.000	.000	.000	.000	.000	.000	.000	.000							
par_9	.000	.000	.000	.000	.000	.000	.000	.000	.000						
par_10	.000	.000	.000	.000	.000	.000	.000	.000	.000	.000					
par_11	.000	.000	.000	.000	.000	.000	.000	.000	.000	.000	.000				
par_12	.000	.000	.000	.000	.000	.000	.000	.000	.000	.000	.000	.000			
par_13	.000	.000	.000	.000	.000	.000	.000	.000	.000	.000	.000	.000	.000		

	par_1	par_2	par_3	par_4	par_5	par_6	par_7	par_8	par_9	par_10	par_11	par_12	par_13	par_14	par_15
par_14	.000	.000	.000	.000	.000	.000	.000	.000	.000	.000	.000	.000	.000	.000	
par_15	.000	.000	.000	.000	.000	.000	.000	.000	.000	.000	.000	.000	.000	.000	.000

	par_1	par_2	par_3	par_4	par_5	par_6	par_7	par_8	par_9	par_10	par_11	par_12	par_13	par_14	par_15
par_1	1.000														
par_2	.506	1.000													
par_3	-.001	.000	1.000												
par_4	.003	-.007	.717	1.000											
par_5	.006	-.008	.645	.687	1.000										
par_6	-.334	-.308	-.466	-.495	-.436	1.000									
par_7	-.608	-.566	.000	.001	.000	.719	1.000								

	par_1	par_2	par_3	par_4	par_5	par_6	par_7	par_8	par_9	par_10	par_11	par_12	par_13	par_14	par_15
par_8	-.002	.004	-.730	-.762	-.682	.799	.226	1.000							
par_9	.238	.203	.000	-.002	.000	-.093	-.227	.001	1.000						
par_10	-.250	-.002	.001	-.010	-.014	.060	.045	.006	-.078	1.000					
par_11	.022	-.200	-.001	.011	.015	.030	.011	-.007	-.019	-.070	1.000				
par_12	.003	-.006	.120	.139	.136	-.073	.001	-.132	-.001	-.009	.010	1.000			
par_13	.009	-.013	-.132	.072	.056	-.007	.001	-.018	-.002	-.021	.023	.024	1.000		
par_14	-.003	.010	.046	-.182	-.013	.047	-.002	.018	.004	.011	-.015	-.025	-.188	1.000	
par_15	-.009	.009	-.001	-.030	-.149	.024	.000	.032	-.001	.019	-.019	-.044	-.089	-.007	1.000

	par_1	par_2	par_3	par_4	par_5	par_6	par_7	par_8	par_9	par_10	par_11	par_12	par_13	par_14	par_15
par_1	.000														
par_2	-2.039	.000													
par_3	14.426	15.457	.000												
par_4	14.571	15.547	-.351	.000											
par_5	7.155	8.226	-11.367	-11.985	.000										
par_6	-30.064	-28.474	-34.140	-34.629	-28.911	.000									
par_7	-16.633	-15.541	-30.010	-30.562	-23.756	15.977	.000								
par_8	-36.558	-34.577	-33.766	-34.215	-28.974	-10.594	-14.688	.000							
par_9	-33.529	-30.734	-35.915	-36.731	-29.881	5.344	-5.401	10.217	.000						

	par_1	par_2	par_3	par_4	par_5	par_6	par_7	par_8	par_9	par_10	par_11	par_12	par_13	par_14	par_15
par_10	-31.931	-32.552	-38.724	-39.564	-32.805	-.134	-10.460	4.950	-5.853	.000					
par_11	-30.994	-26.677	-36.241	-37.220	-30.371	5.255	-6.363	9.862	-.514	5.467	.000				
par_12	-34.249	-31.983	-39.320	-40.512	-33.538	1.811	-9.213	6.762	-4.425	2.173	-3.968	.000			
par_13	-36.094	-33.670	-38.324	-41.141	-34.222	-1.617	-11.419	3.606	-7.739	-1.593	-7.443	-4.060	.000		
par_14	-41.059	-38.938	-42.892	-42.149	-37.003	-9.562	-16.594	-3.574	-15.837	-10.149	-15.529	-13.344	-8.066	.000	
par_15	-34.713	-32.748	-38.671	-39.372	-31.747	.698	-9.956	6.077	-5.554	.904	-5.063	-1.338	2.538	11.864	.000

Model	NPAR	CMIN	DF	P	CMIN/DF
Default model	15	262.413	13	.000	20.186
Saturated model	28	.000	0		
Independence model	7	18593.454	21	.000	885.403

Model	RMR	GFI	AGFI	PGFI
Default model	.014	.986	.969	.458
Saturated model	.000	1.000		
Independence model	.350	.362	.149	.271

Model	NFI Delta1	RFI rho1	IFI Delta2	TLI rho2	CFI
Default model	.986	.977	.987	.978	.987
Saturated model	1.000		1.000		1.000
Independence model	.000	.000	.000	.000	.000

Model	PRATIO	PNFI	PCFI
Default model	.619	.610	.611
Saturated model	.000	.000	.000
Independence model	1.000	.000	.000

Model	NCP	LO 90	HI 90
Default model	249.413	200.450	305.809
Saturated model	.000	.000	.000
Independence model	18572.454	18127.148	19024.036

Model	FMIN	F0	LO 90	HI 90
Default model	.049	.046	.037	.057
Saturated model	.000	.000	.000	.000
Independence model	3.448	3.444	3.361	3.528

Model	RMSEA	LO 90	HI 90	PCLOSE
Default model	.060	.053	.066	.005
Independence model	.405	.400	.410	.000

Model	AIC	BCC	BIC	CAIC
Default model	292.413	292.458	391.309	406.309
Saturated model	56.000	56.083	240.605	268.605
Independence model	18607.454	18607.475	18653.605	18660.605

Model	ECVI	LO 90	HI 90	MECVI
Default model	.054	.045	.065	.054
Saturated model	.010	.010	.010	.010
Independence model	3.450	3.368	3.534	3.450

Model	HOELTER .05	HOELTER .01
Default model	460	570
Independence model	10	12

Minimization: .031

Miscellaneous: .606

Bootstrap: .000

Total: .637

MODEL 4: GENERAL FACTOR AND TWO NESTED FACTORS

Standardized Regression Weights: (Total - Unconstrained)

			Estimate
ph9	<---	General	.662
ph8	<---	General	.653
ph7	<---	General	.713
ph6	<---	General	.802
ph2	<---	General	.842
ph1	<---	General	.722
ph5	<---	General	.663
ph4	<---	General	.703
ph3	<---	General	.666
ph3	<---	F1	.365
ph4	<---	F1	.314
ph5	<---	F1	.338
ph1	<---	F2	.146
ph2	<---	F2	.222
ph6	<---	F2	.032
ph7	<---	F2	-.236
ph8	<---	F2	-.245
ph9	<---	F2	-.027

Model Fit Summary

CMIN

Model	NPAR	CMIN	DF	P	CMIN/DF
Unconstrained	27	315.932	18	.000	17.552
Saturated model	45	.000	0		
Independence model	9	24262.637	36	.000	673.962

RMR, GFI

Model	RMR	GFI	AGFI	PGFI
Unconstrained	.013	.987	.967	.395
Saturated model	.000	1.000		
Independence model	.334	.316	.145	.253

Baseline Comparisons

Model	NFI Delta1	RFI rho1	IFI Delta2	TLI rho2	CFI
Unconstrained	.987	.974	.988	.975	.988
Saturated model	1.000		1.000		1.000
Independence model	.000	.000	.000	.000	.000

Parsimony-Adjusted Measures

Model	PRATIO	PNFI	PCFI
Unconstrained	.500	.493	.494
Saturated model	.000	.000	.000
Independence model	1.000	.000	.000

NCP

Model	NCP	LO 90	HI 90
Unconstrained	297.932	243.967	359.333
Saturated model	.000	.000	.000
Independence model	24226.637	23717.551	24742.000

FMIN

Model	FMIN	F0	LO 90	HI 90
Unconstrained	.059	.055	.045	.067
Saturated model	.000	.000	.000	.000
Independence model	4.499	4.492	4.398	4.588

RMSEA

Model	RMSEA	LO 90	HI 90	PCLOSE
Unconstrained	.055	.050	.061	.046
Independence model	.353	.350	.357	.000

AIC

Model	AIC	BCC	BIC	CAIC
Unconstrained	369.932	370.033	547.944	574.944
Saturated model	90.000	90.167	386.687	431.687
Independence model	24280.637	24280.670	24339.974	24348.974

ECVI

Model	ECVI	LO 90	HI 90	MECVI
Unconstrained	.069	.059	.080	.069
Saturated model	.017	.017	.017	.017
Independence model	4.502	4.408	4.598	4.502

HOELTER

Model	HOELTER .05	HOELTER .01
Unconstrained	493	595
Independence model	12	14

Minimization: .044

Miscellaneous: .582

Bootstrap: .000

Total: .626

MODEL 5: GENERAL FACTOR AND TWO NESTED FACTORS (WITHOUT ITEMS 7 AND 8)

Model Fit Summary

CMIN

Model	NPAR	CMIN	DF	P	CMIN/DF
Default model	22	123.037	6	.000	20.506
Saturated model	28	.000	0		
Independence model	7	18593.454	21	.000	885.403

RMR, GFI

Model	RMR	GFI	AGFI	PGFI
Default model	.010	.993	.969	.213
Saturated model	.000	1.000		
Independence model	.350	.362	.149	.271

Baseline Comparisons

Model	NFI Delta1	RFI rho1	IFI Delta2	TLI rho2	CFI
Default model	.993	.977	.994	.978	.994
Saturated model	1.000		1.000		1.000
Independence model	.000	.000	.000	.000	.000

Parsimony-Adjusted Measures

Model	PRATIO	PNFI	PCFI
Default model	.286	.284	.284
Saturated model	.000	.000	.000
Independence model	1.000	.000	.000

NCP

Model	NCP	LO 90	HI 90
Default model	117.037	84.636	156.871
Saturated model	.000	.000	.000
Independence model	18572.454	18127.148	19024.036

FMIN

Model	FMIN	F0	LO 90	HI 90
Default model	.023	.022	.016	.029
Saturated model	.000	.000	.000	.000
Independence model	3.448	3.444	3.361	3.528

RMSEA

Model	RMSEA	LO 90	HI 90	PCLOSE
Default model	.060	.051	.070	.032
Independence model	.405	.400	.410	.000

AIC

Model	AIC	BCC	BIC	CAIC
Default model	167.037	167.102	312.084	334.084
Saturated model	56.000	56.083	240.605	268.605
Independence model	18607.454	18607.475	18653.605	18660.605

ECVI

Model	ECVI	LO 90	HI 90	MECVI
Default model	.031	.025	.038	.031
Saturated model	.010	.010	.010	.010
Independence model	3.450	3.368	3.534	3.450

HOELTER

Model	HOELTER .05	HOELTER .01
Default model	552	737
Independence model	10	12

Minimization: .085

Miscellaneous:	.542
Bootstrap:	.000
Total:	.627

Estimates (Group number 1 - Default model)

Scalar Estimates (Group number 1 - Default model)

Maximum Likelihood Estimates

Regression Weights: (Group number 1 - Default model)

	Estimate	S.E.	C.R.	P	Label
PHQ9 <--- F1	1.000				
PHQ6 <--- F1	.847	.560	1.514	.130	par_1
PHQ2 <--- F1	.816	.594	1.373	.170	par_2
PHQ1 <--- F1	.630	.556	1.134	.257	par_3
PHQ5 <--- F1	.643	.516	1.245	.213	par_4
PHQ4 <--- F1	.647	.562	1.150	.250	par_5
PHQ3 <--- F1	.652	.566	1.151	.250	par_6
PHQ3 <--- F2	1.000				
PHQ4 <--- F2	.993	.028	35.691	***	par_7
PHQ5 <--- F2	.898	.073	12.376	***	par_8
PHQ1 <--- F3	-2.394	12.534	-.191	.849	par_9
PHQ2 <--- F3	-2.469	13.445	-.184	.854	par_10
PHQ6 <--- F3	-2.266	12.659	-.179	.858	par_11
PHQ9 <--- F3	1.000				

Standardized Regression Weights: (Group number 1 - Default model)

	Estimate
PHQ9 <--- F1	.971
PHQ6 <--- F1	.683
PHQ2 <--- F1	.696
PHQ1 <--- F1	.562
PHQ5 <--- F1	.522
PHQ4 <--- F1	.536
PHQ3 <--- F1	.513
PHQ3 <--- F2	.550
PHQ4 <--- F2	.576
PHQ5 <--- F2	.510
PHQ1 <--- F3	-.490
PHQ2 <--- F3	-.483
PHQ6 <--- F3	-.419

	Estimate
PHQ9 <--- F3	.223

Covariances: (Group number 1 - Default model)

	Estimate	S.E.	C.R.	P	Label
F2 <--> F3	-.067	.221	-.305	.761	par_12

Correlations: (Group number 1 - Default model)

	Estimate
F2 <--> F3	-.798

Variances: (Group number 1 - Default model)

	Estimate	S.E.	C.R.	P	Label
F1	.527	.224	2.355	.019	par_13
F2	.258	.327	.788	.431	par_14
F3	.028	.236	.117	.907	par_15
e1	.004	.262	.015	.988	par_16
e4	.291	.009	33.830	***	par_17
e5	.205	.006	34.078	***	par_18
e6	.294	.007	39.220	***	par_19
e7	.373	.010	36.953	***	par_20
e8	.292	.009	33.912	***	par_21
e9	.369	.010	38.131	***	par_22

Squared Multiple Correlations: (Group number 1 - Default model)

	Estimate
PHQ3	.566
PHQ4	.619
PHQ5	.533
PHQ1	.556
PHQ2	.717
PHQ6	.641
PHQ9	.993

Matrices (Group number 1 - Default model)

Residual Covariances (Group number 1 - Default model)

	PHQ3	PHQ4	PHQ5	PHQ1	PHQ2	PHQ6	PHQ9
PHQ3	.000						
PHQ4	-.008	.000					
PHQ5	.013	-.001	.000				
PHQ1	-.001	.018	-.023	.001			

	PHQ3	PHQ4	PHQ5	PHQ1	PHQ2	PHQ6	PHQ9
PHQ2	-.008	-.001	-.017	.013	.001		
PHQ6	.010	.005	.026	-.016	.001	.001	
PHQ9	.000	-.001	.001	.000	.000	.000	.000

Standardized Residual Covariances (Group number 1 - Default model)

	PHQ3	PHQ4	PHQ5	PHQ1	PHQ2	PHQ6	PHQ9
PHQ3	.006						
PHQ4	-.616	.006					
PHQ5	1.032	-.115	.005				
PHQ1	-.128	1.611	-2.065	.074			
PHQ2	-.612	-.109	-1.421	1.160	.073		
PHQ6	.806	.382	2.084	-1.418	.055	.056	
PHQ9	.030	-.088	.133	-.008	-.006	-.005	.001

	M.I.	Par Change
e8 <--> F3	5.262	-.003
e8 <--> e9	4.712	-.012
e7 <--> F3	6.061	.004
e7 <--> e9	9.377	.018
e6 <--> e8	21.561	.023
e6 <--> e7	27.933	-.028
e5 <--> F3	7.265	-.003
e5 <--> F2	13.657	-.013
e5 <--> e7	13.679	-.017
e5 <--> e6	19.707	.018
e4 <--> F3	9.804	.004
e4 <--> F2	19.411	.018
e4 <--> e7	37.431	.033
e4 <--> e6	25.651	-.024

	M.I.	Par Change

	M.I.	Par Change
PHQ4 <--- PHQ1	10.777	.034
PHQ5 <--- PHQ1	13.777	-.041
PHQ5 <--- PHQ2	4.660	-.023
PHQ5 <--- PHQ6	8.775	.030
PHQ1 <--- PHQ4	6.491	.023
PHQ1 <--- PHQ5	11.092	-.030
PHQ1 <--- PHQ2	4.543	.020
PHQ1 <--- PHQ6	7.762	-.025
PHQ2 <--- PHQ5	9.018	-.024
PHQ2 <--- PHQ1	8.134	.025
PHQ6 <--- PHQ5	22.223	.042
PHQ6 <--- PHQ1	9.727	-.031

Iteration		Negative eigenvalues	Condition #	Smallest eigenvalue	Diameter	F	NTries	Ratio
0	e	6		-1.613	9999.000	19683.598	0	9999.000
1	e	7		-.178	1.304	11927.015	21	.399
2	e	3		-.120	1.192	3548.916	5	.921
3	e	1		-.336	.666	1614.876	5	.631
4	e	1		-.066	.425	690.153	5	.756
5	e	1		-.008	.607	294.700	6	.680
6	e	2		-.004	.298	147.045	5	.800
7	e	0	156775.441		.132	128.533	5	.829
8	e	0	17415.205		.135	128.144	5	.000
9	e	1		-.002	.138	127.344	2	.000
10	e	1		-.002	.090	126.158	8	.876
11	e	1		-.001	.070	125.986	5	.674
12	e	1		-.002	.124	125.724	5	.742
13	e	1		-.001	.074	125.569	4	.579
14	e	1		-.002	.121	125.357	5	.659
15	e	1		.000	.050	125.223	5	.769
16	e	1		-.001	.132	125.053	6	.751
17	e	1		.000	.060	124.938	5	.731
18	e	1		.000	.077	124.821	5	.939
19	e	1		.000	.068	124.744	4	.733
20	e	1		.000	.074	124.645	5	.935
21	e	1		-.001	.074	124.584	4	.603

Iteration		Negative eigenvalues	Condition #	Smallest eigenvalue	Diameter	F	NTries	Ratio
22	e	1		.000	.068	124.495	5	.904
23	e	1		.000	.051	124.443	5	.822
24	e	1		.000	.117	124.365	7	.821
25	e	1		.000	.066	124.306	5	.704
26	e	0	4117542.549		.063	124.247	6	.999
27	e	1		.000	.053	124.210	8	.000
28	e	1		.000	.095	124.147	5	1.149
29	e	0	570075.338		.118	124.090	6	1.155
30	e	1		-.001	.085	124.047	1	.945
31	e	0	4873105.812		.060	123.992	5	.978
32	e	1		.000	.078	123.957	7	.000
33	e	0	705703.461		.069	123.903	5	1.099
34	e	1		.000	.080	123.867	4	.000
35	e	0	736943.634		.058	123.809	4	1.210
36	e	1		.000	.074	123.772	3	.000
37	e	0	650135.901		.077	123.719	5	1.082
38	e	1		.000	.106	123.687	3	.000
39	e	0	1244013.567		.048	123.615	5	1.111
40	e	1		.000	.089	123.574	4	.000
41	e	0	865013.459		.070	123.518	5	1.143
42	e	1		.000	.098	123.475	3	.000
43	e	1		.000	.082	123.412	7	1.233

Iteration		Negative eigenvalues	Condition #	Smallest eigenvalue	Diameter	F	NTries	Ratio
44	e	1		.000	.103	123.364	8	.834
45	e	0	1740838.134		.126	123.297	7	1.234
46	e	1		.000	.123	123.261	2	.000
47	e	0	2970062.251		.078	123.184	5	1.164
48	e	1		.000	.135	123.154	3	.000
49	e	0	47028007.144		.096	123.074	5	1.062

	par_1	par_2	par_3	par_4	par_5	par_6	par_7	par_8	par_9	par_10	par_11	par_12	par_13	par_14	par_15	par_16	par_17	par_18	par_19	par_20	par_21	par_22
par_1	.313																					
par_2	.332	.353																				
par_3	.308	.329	.309																			
par_4	.287	.306	.286	.266																		
par_5	.312	.333	.312	.290	.316																	
pa	.3	.3	.3	.2	.3	.3																

	par_1	par_2	par_3	par_4	par_5	par_6	par_7	par_8	par_9	par_10	par_11	par_12	par_13	par_14	par_15	par_16	par_17	par_18	par_19	par_20	par_21	par_22
r_6	14	35	14	92	18	20																
par_7	.001	.001	.001	.001	.001	.001	.001															
par_8	-.038	-.040	-.037	-.035	-.038	-.038	.000	.005														
par_9	6.119	6.673	6.469	5.913	6.531	6.575	.022	-.771	157.105													
par_10	6.630	7.222	6.989	6.394	7.057	7.104	.024	-.834	168.496	180.779												
par_11	6.276	6.832	6.605	6.045	6.669	6.714	.022	-.788	158.607	170.199	160.259											
par_12	-.101	-.111	-.109	-.099	-.110	-.111	.000	.013	-2.760	-2.955	-2.779	.049										
par	-.0	-.1	-.0	-.0	-.0	-.0	.0	.0	-1.	-1.	-1.	.0	.0									

	par_1	par_2	par_3	par_4	par_5	par_6	par_7	par_8	par_9	par_10	par_11	par_12	par_13	par_14	par_15	par_16	par_17	par_18	par_19	par_20	par_21	par_22
_13	99	00	87	84	88	89	00	11	081	211	166	14	50									
par_14	-.173	-.187	-.178	-.164	-.180	-.181	-.001	.021	-4.038	-4.345	-4.098	.070	.040	.107								
par_15	.113	.123	.120	.109	.121	.122	.000	-.014	2.959	3.171	2.984	-.052	-.018	-.076	.056							
par_16	-.014	-.023	-.033	-.026	-.033	-.033	.000	.003	-1.878	-1.961	-1.818	.038	-.032	.036	-.037	.069						
par_17	.000	.000	.001	.000	.001	.001	.000	.000	.034	.036	.034	-.001	.001	-.001	.001	-.001	.000					
par_18	.000	.000	.000	.000	.000	.000	.000	.000	-.001	-.001	-.001	.000	.000	.000	.000	.000	.000	.000				
par_19	.000	.000	.000	.000	.000	.000	.000	.000	.018	.018	.017	.000	.000	.000	.000	-.001	.000	.000	.000			
par_	.003	.003	.003	.002	.003	.003	.000	.000	.051	.055	.052	-.0	-.0	-.0	.001	.000	.000	.000	.000	.000		

	par_1	par_2	par_3	par_4	par_5	par_6	par_7	par_8	par_9	par_10	par_11	par_12	par_13	par_14	par_15	par_16	par_17	par_18	par_19	par_20	par_21	par_22
20												01	01	01								
par_21	-.002	-.002	-.001	-.001	-.001	-.001	.000	.000	-.028	-.030	-.029	.000	.001	.001	-.001	.000	.000	.000	.000	.000	.000	
par_22	-.001	-.001	-.001	-.001	-.001	-.001	.000	.000	-.021	-.023	-.022	.000	.000	.001	.000	.000	.000	.000	.000	.000	.000	.000

	par_1	par_2	par_3	par_4	par_5	par_6	par_7	par_8	par_9	par_10	par_11	par_12	par_13	par_14	par_15	par_16	par_17	par_18	par_19	par_20	par_21	par_22
par_1	1.000																					
par_2	.998	1.000																				
par_3	.991	.996	1.000																			
par_4	.995	.999	.999	1.000																		
par	.9	.9	1.0	.9	1.0																	

	par_1	par_2	par_3	par_4	par_5	par_6	par_7	par_8	par_9	par_10	par_11	par_12	par_13	par_14	par_15	par_16	par_17	par_18	par_19	par_20	par_21	par_22
_5	92	97	00	99	00																	
par_6	.992	.997	1.000	.999	1.000	1.000																
par_7	.072	.072	.071	.072	.072	.071	1.000															
par_8	-.924	-.927	-.927	-.927	-.927	-.927	.117	1.000														
par_9	.872	.896	.929	.914	.927	.927	.062	-.848	1.000													
par_10	.881	.904	.936	.921	.934	.934	.063	-.855	1.000	1.000												
par_11	.886	.908	.939	.925	.937	.937	.063	-.858	1.000	1.000	1.000											
par_12	-.817	-.845	-.886	-.867	-.883	-.883	-.054	.806	-.995	-.993	-.991	1.000										
par	-.7	-.7	-.6	-.7	-.7	-.7	-.0	.6	-.3	-.4	-.4	.2	1.0									

	par_1	par_2	par_3	par_4	par_5	par_6	par_7	par_8	par_9	par_10	par_11	par_12	par_13	par_14	par_15	par_16	par_17	par_18	par_19	par_20	par_21	par_22
_13	86	54	98	25	02	02	58	74	85	02	11	88	00									
par_14	-.943	-.959	-.978	-.970	-.977	-.977	-.091	.892	-.984	-.987	-.989	.961	.539	1.000								
par_15	.853	.878	.914	.898	.912	.912	.061	-.833	.999	.998	.998	-.998	-.349	-.977	1.000							
par_16	-.097	-.147	-.227	-.190	-.222	-.222	-.006	.175	-.571	-.556	-.547	.652	-.537	.419	-.602	1.000						
par_17	.054	.084	.128	.107	.125	.125	.003	-.098	.319	.311	.307	-.365	.298	-.235	.337	-.558	1.000					
par_18	.001	-.003	-.003	-.001	-.002	-.002	.000	.002	-.012	-.010	-.012	.014	-.016	.007	-.013	.025	-.101	1.000				
par_19	.033	.050	.074	.063	.074	.074	.002	-.058	.189	.182	.180	-.214	.175	-.138	.198	-.327	.149	-.098	1.000			
par_	.458	.458	.455	.456	.455	.455	.041	-.5	.402	.406	.408	-.3	-.3	-.4	.394	-.0	.028	-.0	.016	1.0		

	par_1	par_2	par_3	par_4	par_5	par_6	par_7	par_8	par_9	par_10	par_11	par_12	par_13	par_14	par_15	par_16	par_17	par_18	par_19	par_20	par_21	par_22
20								12				78	57	33		50		01		00		
par_21	-.314	-.312	-.307	-.309	-.308	-.307	-.350	.284	-.259	-.262	-.264	.238	.266	.289	-.252	.000	.000	.000	.000	-.212	1.000	
par_22	-.209	-.208	-.205	-.206	-.205	-.206	.300	.294	-.173	-.175	-.176	.161	.177	.179	-.168	.000	.000	.000	.000	-.144	-.035	1.000

Model	NPAR	CMIN	DF	P	CMIN/DF
Default model	22	123.037	6	.000	20.506
Saturated model	28	.000	0		
Independence model	7	18593.454	21	.000	885.403

Model	RMR	GFI	AGFI	PGFI
Default model	.010	.993	.969	.213
Saturated model	.000	1.000		
Independence model	.350	.362	.149	.271

Model	NFI Delta1	RFI rho1	IFI Delta2	TLI rho2	CFI
Default model	.993	.977	.994	.978	.994
Saturated model	1.000		1.000		1.000
Independence model	.000	.000	.000	.000	.000

Model	PRATIO	PNFI	PCFI
Default model	.286	.284	.284
Saturated model	.000	.000	.000
Independence model	1.000	.000	.000

Model	NCP	LO 90	HI 90
Default model	117.037	84.636	156.871
Saturated model	.000	.000	.000
Independence model	18572.454	18127.148	19024.036

Model	FMIN	F0	LO 90	HI 90
Default model	.023	.022	.016	.029
Saturated model	.000	.000	.000	.000
Independence model	3.448	3.444	3.361	3.528

Model	RMSEA	LO 90	HI 90	PCLOSE
Default model	.060	.051	.070	.032
Independence model	.405	.400	.410	.000

Model	AIC	BCC	BIC	CAIC
Default model	167.037	167.102	312.084	334.084
Saturated model	56.000	56.083	240.605	268.605
Independence model	18607.454	18607.475	18653.605	18660.605

Model	ECVI	LO 90	HI 90	MECVI
Default model	.031	.025	.038	.031
Saturated model	.010	.010	.010	.010
Independence model	3.450	3.368	3.534	3.450

Model	HOELTER .05	HOELTER .01
Default model	552	737
Independence model	10	12

Minimization:	.085
Miscellaneous:	.542
Bootstrap:	.000
Total:	.627

MODEL 6: GENERAL FACTOR AND 3 NESTED FACTORS

Model Fit Summary

CMIN

Model	NPAR	CMIN	DF	P	CMIN/DF
Default model	30	122.771	15	.000	8.185
Saturated model	45	.000	0		
Independence model	9	24262.637	36	.000	673.962

RMR, GFI

Model	RMR	GFI	AGFI	PGFI
Default model	.008	.995	.984	.332
Saturated model	.000	1.000		
Independence model	.334	.316	.145	.253

Baseline Comparisons

Model	NFI Delta1	RFI rho1	IFI Delta2	TLI rho2	CFI
Default model	.995	.988	.996	.989	.996
Saturated model	1.000		1.000		1.000
Independence model	.000	.000	.000	.000	.000

Parsimony-Adjusted Measures

Model	PRATIO	PNFI	PCFI
Default model	.417	.415	.415
Saturated model	.000	.000	.000
Independence model	1.000	.000	.000

NCP

Model	NCP	LO 90	HI 90
Default model	107.771	76.116	146.905
Saturated model	.000	.000	.000
Independence model	24226.637	23717.551	24742.000

FMIN

Model	FMIN	F0	LO 90	HI 90
Default model	.023	.020	.014	.027
Saturated model	.000	.000	.000	.000
Independence model	4.499	4.492	4.398	4.588

RMSEA

Model	RMSEA	LO 90	HI 90	PCLOSE
Default model	.036	.031	.043	1.000
Independence model	.353	.350	.357	.000

AIC

Model	AIC	BCC	BIC	CAIC
Default model	182.771	182.882	380.562	410.562
Saturated model	90.000	90.167	386.687	431.687
Independence model	24280.637	24280.670	24339.974	24348.974

ECVI

Model	ECVI	LO 90	HI 90	MECVI
Default model	.034	.028	.041	.034
Saturated model	.017	.017	.017	.017
Independence model	4.502	4.408	4.598	4.502

HOELTER

Model	HOELTER .05	HOELTER .01
Default model	1098	1344
Independence model	12	14

Minimization: .185
Miscellaneous: .788
Bootstrap: .000
Total: .973

Estimates (Group number 1 - Default model)

Scalar Estimates (Group number 1 - Default model)

Maximum Likelihood Estimates

Regression Weights: (Group number 1 - Default model)

			Estimate	S.E.	C.R.	P	Label
PHQ8	<---	F1	1.000				
PHQ7	<---	F1	1.391	.086	16.190	***	par_1
PHQ9	<---	F1	.839	.090	9.310	***	par_2
PHQ6	<---	F1	1.838	.111	16.608	***	par_3
PHQ2	<---	F1	2.040	.165	12.345	***	par_4
PHQ1	<---	F1	1.987	.219	9.078	***	par_5
PHQ5	<---	F1	1.569	.113	13.934	***	par_6
PHQ4	<---	F1	2.008	.211	9.512	***	par_7
PHQ3	<---	F1	1.878	.170	11.032	***	par_8
PHQ3	<---	F2	.839	.057	14.605	***	par_9
PHQ4	<---	F2	.726	.064	11.332	***	par_10
PHQ5	<---	F2	1.000				
PHQ1	<---	F3	.496	.099	5.027	***	par_11
PHQ2	<---	F3	.828	.093	8.936	***	par_12
PHQ6	<---	F3	1.006	.082	12.299	***	par_13
PHQ9	<---	F3	1.000				
PHQ7	<---	F4	.984	.054	18.365	***	par_14
PHQ8	<---	F4	1.000				

Standardized Regression Weights: (Group number 1 - Default model)

	Estimate
PHQ8 <--- F1	.380
PHQ7 <--- F1	.493
PHQ9 <--- F1	.321
PHQ6 <--- F1	.583
PHQ2 <--- F1	.684
PHQ1 <--- F1	.698
PHQ5 <--- F1	.502
PHQ4 <--- F1	.655
PHQ3 <--- F1	.582
PHQ3 <--- F2	.469
PHQ4 <--- F2	.427
PHQ5 <--- F2	.577
PHQ1 <--- F3	.311
PHQ2 <--- F3	.495
PHQ6 <--- F3	.569
PHQ9 <--- F3	.682
PHQ7 <--- F4	.557
PHQ8 <--- F4	.607

Covariances: (Group number 1 - Default model)

	Estimate	S.E.	C.R.	P	Label
F2 <--> F3	.186	.037	5.031	***	par_15
F2 <--> F4	.175	.032	5.542	***	par_16
F3 <--> F4	.190	.027	7.158	***	par_17

Correlations: (Group number 1 - Default model)

	Estimate
F2 <--> F3	.709
F2 <--> F4	.745
F3 <--> F4	.815

Variances: (Group number 1 - Default model)

	Estimate	S.E.	C.R.	P	Label
F1	.082	.025	3.298	***	par_18
F2	.266	.036	7.411	***	par_19
F3	.260	.030	8.763	***	par_20
F4	.208	.025	8.226	***	par_21
e1	.276	.009	30.521	***	par_22
e2	.291	.008	35.479	***	par_23
e3	.241	.013	19.161	***	par_24

	Estimate	S.E.	C.R.	P	Label
e4	.274	.007	38.228	***	par_25
e5	.208	.006	36.249	***	par_26
e6	.276	.008	32.633	***	par_27
e7	.332	.012	26.844	***	par_28
e8	.298	.009	34.952	***	par_29
e9	.376	.009	41.197	***	par_30

Squared Multiple Correlations: (Group number 1 - Default model)

	Estimate
PHQ3	.558
PHQ4	.612
PHQ5	.584
PHQ1	.583
PHQ2	.714
PHQ6	.663
PHQ9	.568
PHQ7	.553
PHQ8	.513

Matrices (Group number 1 - Default model)

Implied (for all variables) Covariances (Group number 1 - Default model)

	F4	F3	F2	F1	PHQ3	PHQ4	PHQ5	PHQ1	PHQ2	PHQ6	PHQ9	PHQ7	PHQ8
F4	.208												
F3	.190	.260											
F2	.175	.186	.266										
F1	.000	.000	.000	.082									
PHQ3	.147	.156	.223	.153	.851								
PHQ4	.127	.135	.193	.164	.470	.767							
PHQ5	.175	.186	.266	.128	.463	.450	.799						
PHQ1	.094	.129	.093	.162	.382	.393	.347	.663					
PHQ2	.157	.215	.154	.167	.442	.447	.416	.438	.726				
PHQ6	.191	.262	.188	.150	.439	.438	.423	.428	.523	.813			

	F4	F3	F2	F1	PHQ3	PHQ4	PHQ5	PHQ1	PHQ2	PHQ6	PHQ9	PHQ7	PHQ8
PHQ9	.190	.260	.186	.068	.285	.273	.294	.265	.355	.388	.559		
PHQ7	.205	.187	.172	.114	.358	.353	.351	.318	.386	.397	.282	.650	
PHQ8	.208	.190	.175	.082	.300	.291	.303	.256	.324	.341	.258	.319	.566

Implied (for all variables) Correlations (Group number 1 - Default model)

	F4	F3	F2	F1	PHQ3	PHQ4	PHQ5	PHQ1	PHQ2	PHQ6	PHQ9	PHQ7	PHQ8
F4	1.000												
F3	.815	1.000											
F2	.745	.709	1.000										
F1	.000	.000	.000	1.000									
PHQ3	.349	.333	.469	.582	1.000								
PHQ4	.318	.303	.427	.655	.581	1.000							
PHQ5	.430	.409	.577	.502	.562	.575	1.000						
PHQ1	.253	.311	.221	.698	.509	.551	.477	1.000					
PHQ2	.404	.495	.351	.684	.563	.598	.546	.631	1.000				
PHQ6	.464	.569	.404	.583	.528	.554	.525	.583	.681	1.000			
PHQ9	.556	.682	.484	.321	.413	.417	.440	.436	.557	.575	1.000		
PHQ7	.557	.454	.415	.493	.481	.500	.487	.485	.562	.546	.468	1.000	
PHQ8	.607	.495	.452	.380	.433	.442	.451	.419	.505	.503	.459	.525	1.000

Residual Covariances (Group number 1 - Default model)

	PHQ3	PHQ4	PHQ5	PHQ1	PHQ2	PHQ6	PHQ9	PHQ7	PHQ8
PHQ3	.000								
PHQ4	.001	.000							

	PHQ 3	PHQ 4	PHQ 5	PHQ 1	PHQ 2	PHQ 6	PHQ 9	PHQ 7	PHQ 8
PHQ 5	.002	-.002	.000						
PHQ 1	-.006	.000	-.011	.000					
PHQ 2	-.003	-.004	-.007	.010	.000				
PHQ 6	.015	.008	.027	-.013	-.003	.000			
PHQ 9	-.009	.000	-.014	.001	.007	-.004	.000		
PHQ 7	-.008	.008	.008	.001	-.011	.010	-.005	.000	
PHQ 8	.000	-.004	-.005	.012	-.004	-.005	.008	.000	.000

Standardized Residual Covariances (Group number 1 - Default model)

	PHQ 3	PHQ 4	PHQ 5	PHQ 1	PHQ 2	PHQ 6	PHQ 9	PHQ 7	PHQ 8
PHQ 3	.000								
PHQ 4	.049	.000							
PHQ 5	.166	-.197	.000						
PHQ 1	-.516	-.022	-1.039	.000					
PHQ 2	-.251	-.372	-.559	.874	.000				
PHQ 6	1.167	.622	2.186	-1.113	-.221	.000			
PHQ 9	-.845	.018	-1.430	.072	.659	-.352	.000		
PHQ 7	-.689	.766	.709	.111	-1.038	.863	-.498	.000	
PHQ 8	.048	-.449	-.486	1.365	-.394	-.436	.955	.000	.000

			M.I.	Par Change
e6	<-->	F4	8.149	.011
e6	<-->	F2	7.240	-.012
e6	<-->	e7	7.149	-.014
e5	<-->	F4	7.901	-.010
e5	<-->	F3	8.665	.010
e5	<-->	e6	13.857	.015
e4	<-->	F3	12.397	-.013

	M.I.	Par Change
e4 <--> F2	39.552	.028
e4 <--> e9	4.600	.011
e4 <--> e7	30.200	.028
e4 <--> e6	17.803	-.019
e3 <--> F2	12.021	-.015
e3 <--> e7	11.842	-.017
e3 <--> e5	8.073	.011
e2 <--> e9	4.358	-.011
e2 <--> e5	8.316	-.012
e2 <--> e4	6.515	.012
e1 <--> e6	12.052	.016
e1 <--> e4	7.058	-.012
e1 <--> e3	7.638	.013

	M.I.	Par Change

	M.I.	Par Change
PHQ5 <--- PHQ6	8.070	.028
PHQ1 <--- PHQ6	4.935	-.020
PHQ1 <--- PHQ8	6.089	.026
PHQ2 <--- PHQ1	4.950	.020
PHQ2 <--- PHQ7	5.740	-.021
PHQ6 <--- F2	16.728	.091
PHQ6 <--- PHQ3	6.967	.023
PHQ6 <--- PHQ5	22.787	.042
PHQ6 <--- PHQ1	6.143	-.024
PHQ6 <--- PHQ7	4.246	.020
PHQ9 <--- PHQ5	7.523	-.024
PHQ8 <--- PHQ1	4.373	.021

Iteration		Negative eigenvalues	Condition #	Smallest eigenvalue	Diameter	F	NTries	Ratio
0	e	9		-1.452	9999.000	25437.166	0	9999.000
1	e	11		-.128	1.623	14224.084	20	.365
2	e*	4		-.229	1.422	4800.188	4	.772
3	e	1		-.038	.709	1901.227	5	.814
4	e	1		-.120	.808	804.922	6	.791
5	e	0	2736.520		.463	400.797	11	.959
6	e	0	289.522		.262	370.786	5	.000
7	e	1		-.003	.673	235.116	1	.831
8	e	0	734.779		.326	160.934	6	.910

Iteration		Negative eigenvalues	Condition #	Smallest eigenvalue	Diameter	F	NTries	Ratio
9	e	0	1806.720		.314	142.460	2	.000
10	e	0	880.062		.214	135.852	3	.000
11	e	0	1442.426		.271	126.893	1	1.157
12	e	0	2335.426		.242	124.400	1	.988
13	e	0	4146.808		.150	123.109	1	1.105
14	e	0	6009.709		.114	122.841	1	1.165
15	e	0	9167.078		.060	122.779	1	1.173
16	e	0	10929.748		.029	122.771	1	1.099
17	e	0	12056.796		.005	122.771	1	1.024
18	e	0	12024.354		.000	122.771	1	1.001

	par_1	par_2	par_3	par_4	par_5	par_6	par_7	par_8	par_9	par_10	par_11	par_12	par_13	par_14	par_15	par_16	par_17	par_18	par_19	par_20	par_21	par_22	par_23	par_24	par_25	par_26	par_27	par_28	par_29	par_30
par_1	.007																													
par_2	-.002	.008																												
par_3	.008	-.002	.012																											
par	.012	-.0	.017	.027																										

	par_1	par_2	par_3	par_4	par_5	par_6	par_7	par_8	par_9	par_10	par_11	par_12	par_13	par_14	par_15	par_16	par_17	par_18	par_19	par_20	par_21	par_22	par_23	par_24	par_25	par_26	par_27	par_28	par_29	par_30
		05																												
par_4	.016	-.009	.021	.034	.048																									
par_5	.008	-.002	.011	.016	.021	.013																								
par_6	.015	-.008	.020	.033	.044	.021	.045																							
par_7	.012	-.006	.016	.026	.035	.017	.034	.029																						
par_8	.002	-.003	.003	.006	.008	.004	.008	.005	.003																					
par_9	.003	-.004	.004	.008	.011	.015	.010	.008	.003	.004																				
par_10	.006	-.006	.007	.013	.019	.008	.018	.014	.004	.005	.010																			
par_11	.005	-.0	.007	.012	.018	.007	.017	.013	.003	.004	.008	.009																		

	par_1	par_2	par_3	par_4	par_5	par_6	par_7	par_8	par_9	par_10	par_11	par_12	par_13	par_14	par_15	par_16	par_17	par_18	par_19	par_20	par_21	par_22	par_23	par_24	par_25	par_26	par_27	par_28	par_29	par_30
12		04																												
par_13	.004	-.002	.005	.010	.014	.006	.014	.010	.003	.003	.007	.007	.007																	
par_14	.000	-.003	.001	.003	.005	.002	.005	.003	.001	.002	.003	.003	.002	.003																
par_15	.002	-.003	.003	.005	.007	.003	.007	.005	.001	.002	.003	.003	.002	.001	.001															
par_16	.002	-.002	.002	.004	.006	.003	.006	.005	.001	.002	.003	.003	.002	.001	.001	.001														
par_17	.002	-.002	.002	.004	.005	.002	.005	.004	.001	.001	.002	.002	.001	.001	.001	.001	.001													
par_18	-.002	.001	-.002	-.004	-.005	-.002	-.005	-.004	-.001	-.001	-.002	-.002	-.002	-.001	-.001	-.001	-.001	.001												
par_	.002	-.002	.002	.004	.006	.002	.006	.004	.001	.001	.003	.003	.002	.001	.001	.001	.001	-.001	.001											

	par_1	par_2	par_3	par_4	par_5	par_6	par_7	par_8	par_9	par_10	par_11	par_12	par_13	par_14	par_15	par_16	par_17	par_18	par_19	par_20	par_21	par_22	par_23	par_24	par_25	par_26	par_27	par_28	par_29	par_30
19																														
par_20	.001	-.002	.001	.003	.004	.002	.004	.003	.001	.001	.002	.001	.001	.001	.001	.001	.001	-.001	.001	.001										
par_21	.002	-.001	.002	.004	.005	.002	.005	.004	.001	.001	.002	.002	.002	.000	.001	.001	.001	-.001	.001	.000	.001									
par_22	.000	.000	.000	.000	.000	.000	.000	.000	.000	.000	.000	.000	.000	.000	.000	.000	.000	.000	.000	.000	.000	.000								
par_23	.000	.000	.000	.000	.000	.000	.000	.000	.000	.000	.000	.000	.000	.000	.000	.000	.000	.000	.000	.000	.000	.000	.000							
par_24	.000	.000	.000	.001	.001	.000	.001	.001	.000	.000	.000	.001	.001	.000	.000	.000	.000	.000	.000	.000	.000	.000	.000	.000						
par_25	.000	.000	.000	.000	.000	.000	.000	.000	.000	.000	.000	.000	.000	.000	.000	.000	.000	.000	.000	.000	.000	.000	.000	.000	.000					
par_	.000	.000	.000	.000	.000	.000	.000	.000	.000	.000	.000	.000	.000	.000	.000	.000	.000	.000	.000	.000	.000	.000	.000	.000	.000	.000				

	par_1	par_2	par_3	par_4	par_5	par_6	par_7	par_8	par_9	par_10	par_11	par_12	par_13	par_14	par_15
26															
par_27	.000	.000	.000	.000	.000	.000	.000	.000	.000	.000	.000	.000	.000	.000	.000
par_28	.000	.000	.000	.001	.001	.001	.001	.001	.000	.001	.001	.001	.000	.000	.000
par_29	.000	.000	.000	-.001	-.001	.000	-.001	-.001	.000	.000	.000	.000	.000	.000	.000
par_30	.000	.000	.000	.000	.000	.000	.000	.000	.000	.000	.000	.000	.000	.000	.000

	par_16	par_17	par_18	par_19	par_20	par_21	par_22	par_23	par_24	par_25	par_26	par_27	par_28	par_29	par_30
26															
par_27	.000	.000	.000	.000	.000	.000	.000	.000	.000	.000	.000	.000			
par_28	.000	.000	.000	.000	.000	.000	.000	.000	.000	.000	.000	.000	.000		
par_29	.000	.000	.000	.000	.000	.000	.000	.000	.000	.000	.000	.000	.000	.000	
par_30	.000	.000	.000	.000	.000	.000	.000	.000	.000	.000	.000	.000	.000	.000	.000

	par_1	par_2	par_3	par_4	par_5	par_6	par_7	par_8	par_9	par_10	par_11	par_12	par_13	par_14	par_15
par_1	1.000														
par_2	-.220	1.000													

	par_16	par_17	par_18	par_19	par_20	par_21	par_22	par_23	par_24	par_25	par_26	par_27	par_28	par_29	par_30
par_1															
par_2															

	par_1	par_2	par_3	par_4	par_5	par_6	par_7	par_8	par_9	par_10	par_11	par_12	par_13	par_14	par_15	par_16	par_17	par_18	par_19	par_20	par_21	par_22	par_23	par_24	par_25	par_26	par_27	par_28	par_29	par_30
par_3	.835	-.160	1.000																											
par_4	.857	-.347	.903	1.000																										
par_5	.825	-.435	.855	.949	1.000																									
par_6	.812	-.244	.850	.882	.854	1.000																								
par_7	.832	-.438	.851	.945	.952	.880	1.000																							
par_8	.836	-.380	.860	.936	.933	.885	.951	1.000																						
par_9	.485	-.527	.489	.608	.656	.602	.631	.540	1.000																					
par_10	.569	-.619	.573	.714	.772	.658	.707	.697	.798	1.000																				
pa	.6	-.	.6	.8	.8	.7	.8	.8	.6	.7	1.																			

	par_1	par_2	par_3	par_4	par_5	par_6	par_7	par_8	par_9	par_10	par_11	par_12	par_13	par_14	par_15	par_16	par_17	par_18	par_19	par_20	par_21	par_22	par_23	par_24	par_25	par_26	par_27	par_28	par_29	par_30
r_11	67	627	48	21	57	10	87	42	66	81	000																			
par_12	.654	-.495	.638	.786	.874	.698	.866	.825	.632	.743	.928	1.000																		
par_13	.595	-.322	.544	.729	.794	.638	.786	.751	.542	.637	.846	.920	1.000																	
par_14	.099	-.571	.208	.360	.439	.259	.424	.377	.427	.504	.561	.526	.446	1.000																
par_15	.630	-.762	.611	.779	.842	.623	.832	.786	.630	.774	.907	.835	.696	.597	1.000															
par_16	.705	-.597	.706	.855	.905	.711	.897	.860	.610	.753	.931	.907	.813	.480	.947	1.000														
par_17	.691	-.696	.689	.830	.872	.724	.870	.832	.695	.815	.894	.820	.681	.463	.953	.947	1.000													
par	-.8	.5	-.8	-.9	-.9	-.8	-.9	-.9	-.6	-.8	-.9	-.9	-.8	-.4	-.9	-.9	-.9	1.0												

	par_1	par_2	par_3	par_4	par_5	par_6	par_7	par_8	par_9	par_10	par_11	par_12	par_13	par_14	par_15	par_16	par_17	par_18	par_19	par_20	par_21	par_22	par_23	par_24	par_25	par_26	par_27	par_28	par_29	par_30
_18	06	47	20	44	72	40	68	40	84	04	28	02	08	96	04	43	22	00												
par_19	.581	-.594	.567	.722	.783	.503	.765	.725	.400	.574	.839	.815	.727	.530	.915	.917	.822	-.826	1.000											
par_20	.466	-.841	.452	.581	.621	.483	.623	.583	.597	.708	.678	.521	.319	.519	.866	.715	.859	-.701	.661	1.000										
par_21	.751	-.463	.729	.839	.865	.754	.864	.839	.619	.726	.845	.828	.750	.214	.820	.919	.911	-.885	.754	.623	1.000									
par_22	-.118	-.301	-.053	.029	.080	-.023	.069	.042	.142	.169	.177	.152	.112	.552	.219	.119	.119	-.118	.186	.224	-.118	1.000								
par_23	-.025	.246	-.032	-.093	-.128	-.051	-.121	-.101	-.146	-.174	-.188	-.166	-.129	-.409	-.220	-.146	-.146	.156	-.190	-.212	-.097	-.155	1.000							
par_24	.209	.339	.207	.250	.279	.237	.270	.268	.055	.068	.263	.440	.593	.022	.031	.236	.039	-.231	.195	-.372	.251	-.070	.044	1.000						
par_	-.0	-.0	-.0	-.0	-.0	-.0	-.0	-.0	-.0	-.0	-.0	-.1	-.2	-.0	.008	-.0	.010	.051	-.0	.110	-.0	.018	-.0	-.2	1.0					

	par_1	par_2	par_3	par_4	par_5	par_6	par_7	par_8	par_9	par_10	par_11	par_12	par_13	par_14	par_15	par_16	par_17	par_18	par_19	par_20	par_21	par_22	par_23	par_24	par_25	par_26	par_27	par_28	par_29	par_30
25	48	91	37	54	64	54	62	61	09	11	60	03	26	02		52			42		56		11	42	00					
par_26	-.039	.043	-.051	-.124	-.042	-.039	-.046	-.046	-.052	-.066	-.062	-.028	-.025	-.036	-.056	-.064	-.058	.060	-.063	-.059	-.056	-.008	.005	.013	-.072	1.000				
par_27	-.061	-.007	-.073	-.080	-.220	-.057	-.068	-.067	-.119	-.151	-.008	-.157	-.162	-.085	-.089	-.118	-.076	.109	-.121	-.001	-.096	-.031	.021	-.142	.030	-.100	1.000			
par_28	.355	-.384	.360	.443	.473	.485	.463	.434	.661	.646	.486	.454	.385	.304	.440	.429	.510	-.499	.204	.443	.454	.095	-.099	.023	-.003	-.023	-.040	1.000		
par_29	-.302	.291	-.294	-.369	-.390	-.332	-.455	-.387	-.307	-.356	-.452	-.430	-.386	-.263	-.408	-.419	-.417	.428	-.375	-.323	-.389	-.083	.085	-.114	.028	-.003	-.025	-.246	1.000	
par_30	-.087	.091	-.087	-.108	-.115	-.105	-.114	-.122	-.226	-.124	-.123	-.116	-.100	-.075	-.105	-.103	-.124	.123	-.078	-.104	-.112	-.024	.024	-.014	.003	.004	.007	-.134	.019	1.000

	par_1	par_2	par_3	par_4	par_5	par_6	par_7	par_8	par_9	par_10	par_11	par_12	par_13	par_14	par_15	par_16	par_17	par_18	par_19	par_20	par_21	par_22	par_23	par_24	par_25	par_26	par_27	par_28	par_29	par_30
par_1	.000																													

	par_1	par_2	par_3	par_4	par_5	par_6	par_7	par_8	par_9	par_10	par_11	par_12	par_13	par_14	par_15	par_16	par_17	par_18	par_19	par_20	par_21	par_22	par_23	par_24	par_25	par_26	par_27	par_28	par_29	par_30
par_2	-4.021	.000																												
par_3	7.291	6.514	.000																											
par_4	6.369	5.617	2.490	.000																										
par_5	3.824	4.246	1.087	-.645	.000																									
par_6	2.689	4.552	-4.406	-5.559	-3.076	.000																								
par_7	4.178	4.441	1.298	-.410	.309	3.539	.000																							
par_8	4.457	4.707	.421	-2.693	-1.273	3.512	-1.807	.000																						
par_9	-7.1	.003	-10.3	-8.6	-6.1	-8.0	-6.4	-7.0	.000																					

	par_1	par_2	par_3	par_4	par_5	par_6	par_7	par_8	par_9	par_10	par_11	par_12	par_13	par_14	par_15	par_16	par_17	par_18	par_19	par_20	par_21	par_22	par_23	par_24	par_25	par_26	par_27	par_28	par_29	par_30
	94		41	98	61	66	78	48																						
par_10	-9.211	-.810	-12.261	-10.296	-7.237	-9.874	-7.460	-8.615	-2.895	.000																				
par_11	-11.741	-2.010	-15.168	-15.227	-10.384	-13.160	-11.478	-13.534	-4.625	-3.642	.000																			
par_12	-7.565	-.069	-11.479	-11.155	-7.987	-9.050	-8.505	-9.777	-.158	1.638	9.009	.000																		
par_13	-5.102	1.196	-8.730	-8.651	-6.067	-6.447	-6.451	-7.181	2.383	4.366	9.672	4.895	.000																	
par_14	-4.218	1.130	-7.595	-6.844	-4.986	-5.249	-5.649	-5.659	2.433	4.351	5.965	1.974	-.294	.000																
par_15	-17.487	-5.401	-17.802	-13.399	-9.540	-14.692	-10.040	-11.834	-14.622	-12.713	-4.627	-9.873	-13.220	-18.422	.000															

	par_1	par_2	par_3	par_4	par_5	par_6	par_7	par_8	par_9	par_10	par_11	par_12	par_13	par_14	par_15	par_16	par_17	par_18	par_19	par_20	par_21	par_22	par_23	par_24	par_25	par_26	par_27	par_28	par_29	par_30
par_16	-18.019	-5.928	-18.241	-13.400	-9.499	-15.013	-9.999	-11.828	-14.545	-12.155	-4.571	-9.987	-14.069	-17.064	-.898	.000														
par_17	-17.098	-5.890	-17.464	-12.848	-9.160	-14.488	-9.646	-11.338	-14.947	-11.876	-4.035	-8.799	-12.249	-16.708	.227	1.339	.000													
par_18	-12.250	-9.547	-13.331	-10.373	-7.840	-11.091	-8.192	-9.274	-9.892	-7.557	-3.396	-6.461	-8.987	-13.021	-1.735	-1.684	-2.149	.000												
par_19	-15.778	-4.981	-16.541	-12.534	-8.961	-13.097	-9.412	-11.018	-10.581	-8.776	-3.231	-8.425	-12.148	-15.605	5.258	6.301	3.683	3.171	.000											
par_20	-14.743	-4.981	-15.653	-11.868	-8.557	-12.878	-9.010	-10.450	-12.500	-9.665	-2.895	-6.989	-9.610	-15.790	3.939	3.654	4.158	3.519	-.199	.000										
par_21	-17.149	-6.047	-17.369	-12.663	-9.011	-14.328	-9.489	-11.158	-13.633	-10.589	-3.668	-8.480	-12.273	-14.329	.997	2.531	1.682	2.603	-2.431	-2.145	.000									

	par_1	par_2	par_3	par_4	par_5	par_6	par_7	par_8	par_9	par_10	par_11	par_12	par_13	par_14	par_15	par_16	par_17	par_18	par_19	par_20	par_21	par_22	par_23	par_24	par_25	par_26	par_27	par_28	par_29	par_30
par_22	-12.756	-6.041	-14.011	-10.677	-7.837	-11.427	-8.223	-9.420	-9.905	-7.127	-2.261	-6.020	-8.984	-14.403	2.467	3.152	3.189	7.095	.286	.537	2.421	.000								
par_23	-12.721	-6.198	-13.911	-10.525	-7.708	-11.280	-8.091	-9.268	-9.262	-6.596	-2.044	-5.693	-8.592	-12.073	2.626	3.411	3.491	8.411	.655	.942	3.013	1.141	.000							
par_24	-13.655	-6.894	-14.680	-11.064	-8.092	-12.034	-8.490	-9.781	-10.279	-7.517	-2.648	-6.675	-10.190	-13.555	1.419	2.123	1.792	5.279	-.677	-.513	1.315	-2.137	-3.338	.000						
par_25	-12.908	-6.206	-14.071	-10.653	-7.806	-11.439	-8.193	-9.390	-9.752	-7.005	-2.237	-5.915	-8.746	-13.132	2.318	3.004	3.027	7.557	.223	.461	2.458	-.163	-1.536	2.030	.000					
par_26	-13.705	-7.008	-14.672	-11.033	-8.116	-12.048	-8.513	-9.790	-10.876	-8.007	-2.906	-6.667	-9.716	-14.347	.566	1.001	.661	5.031	-1.576	-1.710	-.014	-6.318	-8.302	-2.442	-6.952	.000				
par_27	-12.839	-6.214	-13.996	-10.617	-7.746	-11.401	-8.176	-9.367	-9.534	-6.830	-2.221	-5.849	-8.733	-12.883	2.311	2.989	3.036	7.688	.275	.515	2.469	.026	-1.256	2.138	.200	6.363	.000			

	par_1	par_2	par_3	par_4	par_5	par_6	par_7	par_8	par_9	par_10	par_11	par_12	par_13	par_14	par_15	par_16	par_17	par_18	par_19	par_20	par_21	par_22	par_23	par_24	par_25	par_26	par_27	par_28	par_29	par_30
par_28	-12.856	-5.299	-14.093	-10.662	-7.757	-11.540	-8.147	-9.353	-10.106	-6.921	-1.755	-5.647	-8.649	-12.725	4.356	5.494	6.254	7.657	1.886	2.717	5.503	3.882	2.695	5.206	4.091	9.048	3.672	.000		
par_29	-12.303	-6.147	-13.574	-10.334	-7.597	-10.985	-7.950	-9.096	-8.929	-6.336	-1.929	-5.485	-8.287	-12.159	2.697	3.398	3.483	9.614	.808	1.287	3.071	1.711	.633	3.520	2.184	8.747	1.792	-2.078	.000	
par_30	-11.643	-5.157	-13.073	-9.995	-7.319	-10.471	-7.686	-8.753	-7.694	-5.315	-1.199	-4.799	-7.570	-11.047	4.849	5.935	6.407	11.625	2.931	3.628	6.024	7.242	7.047	8.587	8.821	15.627	8.063	2.667	6.323	.000

Model	NPAR	CMIN	DF	P	CMIN/DF
Default model	30	122.771	15	.000	8.185
Saturated model	45	.000	0		
Independence model	9	24262.637	36	.000	673.962

Model	RMR	GFI	AGFI	PGFI
Default model	.008	.995	.984	.332
Saturated model	.000	1.000		
Independence model	.334	.316	.145	.253

Model	NFI Delta1	RFI rho1	IFI Delta2	TLI rho2	CFI
Default model	.995	.988	.996	.989	.996
Saturated model	1.000		1.000		1.000
Independence model	.000	.000	.000	.000	.000

Model	PRATIO	PNFI	PCFI
Default model	.417	.415	.415
Saturated model	.000	.000	.000
Independence model	1.000	.000	.000

Model	NCP	LO 90	HI 90
Default model	107.771	76.116	146.905
Saturated model	.000	.000	.000

Model	NCP	LO 90	HI 90
Independence model	24226.637	23717.551	24742.000

Model	FMIN	F0	LO 90	HI 90
Default model	.023	.020	.014	.027
Saturated model	.000	.000	.000	.000
Independence model	4.499	4.492	4.398	4.588

Model	RMSEA	LO 90	HI 90	PCLOSE
Default model	.036	.031	.043	1.000
Independence model	.353	.350	.357	.000

Model	AIC	BCC	BIC	CAIC
Default model	182.771	182.882	380.562	410.562
Saturated model	90.000	90.167	386.687	431.687
Independence model	24280.637	24280.670	24339.974	24348.974

Model	ECVI	LO 90	HI 90	MECVI
Default model	.034	.028	.041	.034
Saturated model	.017	.017	.017	.017
Independence model	4.502	4.408	4.598	4.502

Model	HOELTER .05	HOELTER .01
Default model	1098	1344
Independence model	12	14

Minimization:	.185
Miscellaneous:	.788
Bootstrap:	.000
Total:	.973

FACTORIAL INVARIANCE

SEX

Model Fit Summary

CMIN

Model	NPAR	CMIN	DF	P	CMIN/DF
Unconstrained	60	140.521	30	.000	4.684
Model 1	46	182.527	44	.000	4.148
Model 2	39	220.468	51	.000	4.323
Model 3	30	262.937	60	.000	4.382
Model 4	30	262.937	60	.000	4.382
Saturated model	90	.000	0		
Independence model	18	24159.107	72	.000	335.543

RMR, GFI

Model	RMR	GFI	AGFI	PGFI
Unconstrained	.008	.994	.982	.331
Model 1	.014	.992	.984	.485
Model 2	.039	.991	.984	.561
Model 3	.042	.989	.984	.659
Model 4	.042	.989	.984	.659
Saturated model	.000	1.000		
Independence model	.329	.318	.147	.254

Baseline Comparisons

Model	NFI Delta1	RFI rho1	IFI Delta2	TLI rho2	CFI
Unconstrained	.994	.986	.995	.989	.995
Model 1	.992	.988	.994	.991	.994
Model 2	.991	.987	.993	.990	.993
Model 3	.989	.987	.992	.990	.992
Model 4	.989	.987	.992	.990	.992
Saturated model	1.000		1.000		1.000
Independence model	.000	.000	.000	.000	.000

Parsimony-Adjusted Measures

Model	PRATIO	PNFI	PCFI
Unconstrained	.417	.414	.415
Model 1	.611	.606	.608
Model 2	.708	.702	.703
Model 3	.833	.824	.826
Model 4	.833	.824	.826
Saturated model	.000	.000	.000
Independence model	1.000	.000	.000

NCP

Model	NCP	LO 90	HI 90
Unconstrained	110.521	77.365	151.217
Model 1	138.527	100.566	184.048
Model 2	169.468	127.254	219.235

Model	NCP	LO 90	HI 90
Model 3	202.937	156.452	256.972
Model 4	202.937	156.452	256.972
Saturated model	.000	.000	.000
Independence model	24087.107	23579.309	24601.187

FMIN

Model	FMIN	F0	LO 90	HI 90
Unconstrained	.026	.020	.014	.028
Model 1	.034	.026	.019	.034
Model 2	.041	.031	.024	.041
Model 3	.049	.038	.029	.048
Model 4	.049	.038	.029	.048
Saturated model	.000	.000	.000	.000
Independence model	4.481	4.467	4.373	4.563

RMSEA

Model	RMSEA	LO 90	HI 90	PCLOSE
Unconstrained	.026	.022	.031	1.000
Model 1	.024	.021	.028	1.000
Model 2	.025	.022	.028	1.000
Model 3	.025	.022	.028	1.000
Model 4	.025	.022	.028	1.000
Independence model	.249	.246	.252	.000

AIC

Model	AIC	BCC	BIC	CAIC
Unconstrained	260.521	260.972		
Model 1	274.527	274.873		
Model 2	298.468	298.762		
Model 3	322.937	323.162		
Model 4	322.937	323.162		
Saturated model	180.000	180.677		
Independence model	24195.107	24195.243		

ECVI

Model	ECVI	LO 90	HI 90	MECVI
Unconstrained	.048	.042	.056	.048
Model 1	.051	.044	.059	.051
Model 2	.055	.048	.065	.055
Model 3	.060	.051	.070	.060
Model 4	.060	.051	.070	.060
Saturated model	.033	.033	.033	.034
Independence model	4.487	4.393	4.583	4.487

HOELTER

Model	HOELTER .05	HOELTER .01
Unconstrained	1681	1954
Model 1	1788	2031
Model 2	1681	1894
Model 3	1623	1814
Model 4	1623	1814
Independence model	22	24

Model	DF	CMIN	P	NFI Delta-1	IFI Delta-2	RFI rho-1	TLI rho2
Model 1	14	42.006	.000	.002	.002	-.002	-.002
Model 2	21	79.947	.000	.003	.003	-.001	-.001
Model 3	30	122.416	.000	.005	.005	-.001	-.001
Model 4	30	122.416	.000	.005	.005	-.001	-.001

Model	DF	CMIN	P	NFI Delta-1	IFI Delta-2	RFI rho-1	TLI rho2
Model 2	7	37.942	.000	.002	.002	.001	.001
Model 3	16	80.410	.000	.003	.003	.001	.001
Model 4	16	80.410	.000	.003	.003	.001	.001

Model	DF	CMIN	P	NFI Delta-1	IFI Delta-2	RFI rho-1	TLI rho2
Model 3	9	42.468	.000	.002	.002	.000	.000
Model 4	9	42.468	.000	.002	.002	.000	.000

Minimization:	.080
Miscellaneous:	1.857
Bootstrap:	.000
Total:	1.937

FACTORIAL INVARIANCE

AGE

Model Fit Summary

CMIN

Model	NPAR	CMIN	DF	P	CMIN/DF
Unconstrained	60	139.236	30	.000	4.641
Model 1	46	158.259	44	.000	3.597
Model 2	39	194.561	51	.000	3.815
Model 3	30	366.790	60	.000	6.113
Model 4	30	366.790	60	.000	6.113
Saturated model	90	.000	0		
Independence model	18	24267.290	72	.000	337.046

RMR, GFI

Model	RMR	GFI	AGFI	PGFI
Unconstrained	.008	.994	.982	.331
Model 1	.010	.993	.986	.486
Model 2	.018	.992	.986	.562
Model 3	.025	.985	.978	.657
Model 4	.025	.985	.978	.657
Saturated model	.000	1.000		
Independence model	.331	.317	.146	.253

Baseline Comparisons

Model	NFI Delta1	RFI rho1	IFI Delta2	TLI rho2	CFI
Unconstrained	.994	.986	.995	.989	.995
Model 1	.993	.989	.995	.992	.995
Model 2	.992	.989	.994	.992	.994
Model 3	.985	.982	.987	.985	.987
Model 4	.985	.982	.987	.985	.987
Saturated model	1.000		1.000		1.000
Independence model	.000	.000	.000	.000	.000

Parsimony-Adjusted Measures

Model	PRATIO	PNFI	PCFI
Unconstrained	.417	.414	.415
Model 1	.611	.607	.608
Model 2	.708	.703	.704
Model 3	.833	.821	.823
Model 4	.833	.821	.823
Saturated model	.000	.000	.000
Independence model	1.000	.000	.000

NCP

Model	NCP	LO 90	HI 90
Unconstrained	109.236	76.270	149.744
Model 1	114.259	79.588	156.519
Model 2	143.561	104.517	190.183
Model 3	306.790	250.123	370.960
Model 4	306.790	250.123	370.960
Saturated model	.000	.000	.000
Independence model	24195.290	23686.347	24710.514

FMIN

Model	FMIN	F0	LO 90	HI 90
Unconstrained	.026	.020	.014	.028
Model 1	.029	.021	.015	.029
Model 2	.036	.027	.019	.035
Model 3	.068	.057	.046	.069
Model 4	.068	.057	.046	.069
Saturated model	.000	.000	.000	.000
Independence model	4.501	4.487	4.393	4.583

RMSEA

Model	RMSEA	LO 90	HI 90	PCLOSE
Unconstrained	.026	.022	.030	1.000
Model 1	.022	.018	.026	1.000
Model 2	.023	.019	.026	1.000
Model 3	.031	.028	.034	1.000

Model	RMSEA	LO 90	HI 90	PCLOSE
Model 4	.031	.028	.034	1.000
Independence model	.250	.247	.252	.000

AIC

Model	AIC	BCC	BIC	CAIC
Unconstrained	259.236	259.691		
Model 1	250.259	250.608		
Model 2	272.561	272.857		
Model 3	426.790	427.018		
Model 4	426.790	427.018		
Saturated model	180.000	180.682		
Independence model	24303.290	24303.426		

ECVI

Model	ECVI	LO 90	HI 90	MECVI
Unconstrained	.048	.042	.056	.048
Model 1	.046	.040	.054	.046
Model 2	.051	.043	.059	.051
Model 3	.079	.069	.091	.079
Model 4	.079	.069	.091	.079
Saturated model	.033	.033	.033	.034
Independence model	4.507	4.413	4.603	4.507

HOELTER

Model	HOELTER .05	HOELTER .01
Unconstrained	1697	1972
Model 1	2062	2342
Model 2	1905	2146
Model 3	1164	1301
Model 4	1164	1301
Independence model	22	24

Model	DF	CMIN	P	NFI Delta-1	IFI Delta-2	RFI rho-1	TLI rho2
Model 1	14	19.023	.164	.001	.001	-.003	-.003
Model 2	21	55.326	.000	.002	.002	-.002	-.002

Model 3	30	227.554	.000	.009	.009	.004	.004
Model 4	30	227.554	.000	.009	.009	.004	.004

Model	DF	CMIN	P	NFI Delta-1	IFI Delta-2	RFI rho-1	TLI rho2
Model 2	7	36.302	.000	.001	.001	.001	.001
Model 3	16	208.531	.000	.009	.009	.007	.007
Model 4	16	208.531	.000	.009	.009	.007	.007

Model	DF	CMIN	P	NFI Delta-1	IFI Delta-2	RFI rho-1	TLI rho2
Model 3	9	172.229	.000	.007	.007	.007	.007
Model 4	9	172.229	.000	.007	.007	.007	.007

Minimization:	.085
Miscellaneous:	1.842
Bootstrap:	.000
Total:	1.927
